# Optimized Combinations of Filtrates of *Trichoderma* spp., *Metarhizium* spp., and *Bacillus* spp. in the Biocontrol of Rice Pests and Diseases

**DOI:** 10.3390/jof11070471

**Published:** 2025-06-20

**Authors:** Xifen Zhang, Lusheng Chen, Zhenxu Bai, Yaqian Li, Jie Chen

**Affiliations:** 1 School of Agriculture and Biology, Shanghai Jiao Tong University, Shanghai 200240, China; 2 The State Key Laboratory of Microbial Metabolism, Shanghai Jiao Tong University, Shanghai 200240, China

**Keywords:** microbial metabolic fluids, rice pests and diseases, biocontrol metabolites, plant immunity

## Abstract

*Trichoderma* spp., *Metarhizium* spp., and *Bacillus* spp. are commonly used as biocontrol microorganisms domestically and internationally. However, microbial pesticides currently prepared from single living microorganisms have problems such as a short shelf life, particularly under stressful environment conditions. Secondary metabolites produced from biocontrol microorganisms are comparatively stable when used under field conditions. This study screened the optimal combination of biocontrol metabolites, referred to as TMB, composed of culture filtrates from certain isolates of *Trichoderma asperellum* 10264, *Bacillus subtilis* S4-4-10, and *Metarhizium anisopliae* 3.11962 (1:4:1 (*v*/*v*)). RNA-seq analysis and transmission electron microscope observations were carried out to identify the major functions of the most effective culture filtrates against *Magnaporthe oryzae* (the pathogen causing rice blast disease) and *Chilo suppressalis* (an insect pest in rice cultivation). TMB was found to disrupt the midgut subcellular structure of *C. suppressalis* larvae and inhibit the expression of genes related to immunity, membrane components, protein synthesis, and other functions in *C. suppressalis* larvae and *M. oryzae*, thereby interfering with their normal growth, reproduction, and infection potential in rice. In addition, TMB was also able to promote rice growth and trigger host defense responses against infections by the target pests and pathogens. In summary, TMB generated different inhibitory activities against multiple targets in *C. suppressalis* and *M. oryzae* and induced plant immunity in rice. Therefore, it can be used as a new environmentally friendly agent or alternative to control rice pests and diseases.

## 1. Introduction

Rice is one of the most important crops in the world [1,2], but it suffers from various diseases and pest insects during its growth season. These include fungal diseases, such as rice blast disease caused by *Magnaporthe oryzae* [3] and rice sheath blight caused by *Rhizoctonia solani* [4], as well as pest insects, such as *Chilo suppressalis* [5,6], all of which result in serious reductions in grain yield and quality. At present, the prevention and control of rice diseases and pest insects mainly rely on the use of chemical agents. Unfortunately, their unreasonable utilization tends to result in environmental pollution and pathogen and pest resistance [7,8,9].

Biological microorganisms and their metabolites are more environmentally friendly for both the environment and human health when compared to chemical pesticides [10]. *Trichoderma* spp., *Metarhizium* spp., and *Bacillus* spp. have been found to be effective in controlling rice pests and diseases [11,12,13]. de Sousa TP et al. [11] reported that *T. asperellum* significantly inhibited the growth of rice blast fungus (paired -91.18% by *T. asperellum* Ufra.T09) and reduced leaf blast severity by 94.64% (*T. asperellum* Ufra.T52cs) in a simultaneous application and by 85% (*T. asperellum* Ufra.T09 24 and *T. asperellum* 48 hasi) in a curative application in vivo. Shahriari M et al. have shown that the isolates of *B. bassiana*, *M. anisopliae*, and *H. subulata* cause high mortality in *C. suppressalis* [12]. The antifungal metabolites produced by *Bacillus siamensis* L34, *B. amyloliquefaciens* RA37, *B. velezensis* L12, and *B. subtilis* B18 can target *M. oryzae* and also induce the systemic resistance of plants [14]. This implies that culture filtrates prepared from biocontrol microbes might be an alternative for the control of plant disease and pest insects more efficiently, since a large amount of evidence has revealed that live microorganisms are commonly challenged by adverse field conditions, thereby leading to unstable control efficacy. Therefore, culture filtrate prepared from biocontrol microbes are among the most practical alternatives to realize stable and effective control of crop diseases and pest insects [14,15]. Culture filtrates of biocontrol agents, used as adjuvants, have gained increasing acceptance. This is mainly because the cost of developing new active ingredients is still much higher than the cost of the development of new adjuvants. *B. subtilis* KLBMPGC81 (KC81) and its supernatant (SUP) can suppress the growth, appressorium formation, and host infection of *M. oryzae*. The SUP abnormally activates the pathogen’s cell wall integrity pathway and disrupts the cytoskeleton, mitosis, and autophagy [16]. Kgosi VT et al. found that sterilized culture filtrate of *B. subtilis* DL76 can delay and even suppress the germination of conidia and prevent the formation of appressorium in vitro and in vivo [17].

To date, research on the application of biocontrol stain metabolites has mainly focused on developing biopesticides containing only one or two highly purified functional components, targeting only a very narrow spectrum of plant diseases and pest insects, while being easy to decompose [18]. Therefore, metabolites from different microbial isolates are urgently needed. Unfortunately, so far, little work has been conducted in this direction for the biocontrol of rice diseases and pest insects.

To solve this problem, this study screened and optimized a combination of metabolic fluids derived from *T. asperellum* 10264, *B. subtilis* S4-4-10, and *M. anisopliae* 3.11962 (TMB) both in vitro and in plants. The results show that TMB more efficiently inhibited the growth and reproduction of *R. solani*, *M. oryzae*, and *C. suppressalis*. Additionally, rice immune responses were also triggered by TMB against the pests. The physiological and gene expression changes caused by TMB in the three pathogens and pest insects were detected using different approaches, such as transmission electron microscopy (TEM) and transcriptome analysis. The synergistic mechanism of TMB relative to a single microbial filtrate against diseases and pests was explored. Our research findings establish a better foundation for the biocontrol of plant diseases and pest insects.

## 2. Materials and Methods

### 2.1. Strains, Insects, and Plants

*Trichoderma asperellum* CTCCSJ-W-SBW 10264, *Metarhizium anisopliae* 3.11962, *Rhizotonia solani*, and *Magnaporthe oryzae* were cultured at 28 °C on potato dextrose agar (PDA) medium (Qingdao Hope Bio-Technology Co., Ltd., Qingdao, China) for 7 days. *Bacillus subtilis* S4-4-10 was cultured on Luria-Bertani agar (LA) medium (Qingdao Hope Bio-Technology Co., Ltd., Qingdao, China) at 28 °C for 2 days. The above strains were all preserved at the Culture Preservation Center of Shanghai Jiao Tong University. *Chilo suppressalis* (2–3 instars) was purchased from Jilin Haokang Biotechnology (Jilin, China). Rice (*Oryza sativa* L.) Nangeng 46 was provided by the Jinshan District Agricultural Technology Extension Center, Shanghai, China.

### 2.2. Single-Factor Optimization Experiment for Culture Medium Components

Potato dextrose medium was selected as the initial medium for culturing *T. asperellum* 10264 and *M. anisopliae* 3.11962, and LB medium was selected as the initial medium for culturing *B. subtilis* S4-4-10. To identify the optimal carbon sources, sucrose (A), corn flour (B), maltose (C), molasses (D), and galactose (E) were selected to replace the carbon source in the initial medium. Similarly, yeast powder (F), peptone (G), soy peptone (H), beef extract (I), and skim milk powder (J) were used to replace the nitrogen source in the initial medium. Ammonium sulfate (K), ferrous sulfate heptahydrate (L), magnesium sulfate heptahydrate (M), potassium hydrogen phosphate (N), and potassium dihydrogen phosphate (O) were used to replace the inorganic salts in the original medium. All of the above reagents were purchased from Sinopharm Group Co., Ltd. (Shanghai, China). The spore suspensions (1 × 10^6^ spores/mL, 1 mL) of *T. asperellum* 10264 and *M. anisopliae* 3.11962 were inoculated into media with different components (100 mL/250 mL flask) and cultured for 5 d (28 °C and 200 rpm). *B. subtilis* S4-4-10 cultures (*OD_600_* = 0.8, 1 mL) were inoculated into LB medium and cultured for 5 d (28 °C and 200 rpm). After centrifuging the above culture at 8000 rpm/min for 10 min, supernatants were filtered through a 0.22 μm membrane to obtain sterile culture filtrates.

The inhibitory activity of the sterile culture filtrate against *R. solani* and *M. oryzae* was estimated using the mycelial growth rate method [19]. Briefly, the culture filtrate was added to PDA medium (40 °C, 1:3 (*V*/*V*)). Mycelial plugs (5-mm diameter) were cut from the edge of a 7-day-old colony, inoculated in the center of PDA plates, and then incubated at 28 °C for 7 days. This experiment was conducted with three biological replicates. The mycelial diameter was measured using the cross method to calculate the inhibition rate [20]. The inhibition rate was calculated as [(R − r)/R] × 100, where R and r are the average diameters of the control and treatment colonies, respectively. Single-factor optimization of the culture medium was carried out based on the screening criteria for the inhibitory rates of metabolic fluid against *R. solani* and *M. oryzae*.

### 2.3. Plackett–Burman Design

The Plackett–Burman (PB) design was used to investigate the composition of the medium identified through a single-factor experiment consisting of 15 trials, which included 12 factor screening trials and 3 block center points [21].

For *T. asperellum* 10264, the factors maltose (A), galactose (B), skim milk powder (C), ammonium sulfate (D), magnesium sulfate (E), dipotassium hydrogen phosphate (F), and blank (G, H) were each tested at the three levels of 1, 0, and −1 (Table S1). Three parallel experiments were carried out for each group. The inoculation, cultivation, and antifungal effects of the culture filtrate of *T. asperellum* 10264 were the same as those described above. The most significant medium components among those mentioned above were determined based on the inhibition rates of culture filtrate against *R. solani* and *M. oryzae*, as well as *C. suppressalis*.

For *B. subtilis* S4-4-10, A–H represent molasses, galactose, yeast powder, beef extract, ammonium sulfate, magnesium sulfate heptahydrate, potassium dihydrogen phosphate, and blank, respectively (Table S2). The experimental method and screening criteria were the same as those mentioned above.

For *M. anisopliae* 3.11962, A–H represent sucrose, molasses, soy peptone, beef extract, ammonium sulfate, magnesium sulfate heptahydrate, potassium dihydrogen phosphate, potassium dihydrogen phosphate, and blank, respectively (Table S3). The experimental method and screening criteria were the same as those mentioned above.

### 2.4. Central Composite Design

The coefficient values of the four most significant influencing factors obtained through the PB design were used to determine the climbing direction and step size of the steepest climbing test, and then the four most significant influencing factors and their concentrations were determined. Using this as the center point of the response surface, we designed a 4-factor 5-level experiment with 31 iterations (Tables S4 and S5). Each experiment was repeated three times and subjected to response surface analysis. We analyzed the significance of the response coefficients for different variables, optimized the model, and obtained the optimal conditions for prediction. Subsequently, 5 parallel experiments were conducted under optimal conditions to analyze whether the actual results were within the 95% confidence interval of the predicted values in order to verify the accuracy of the model predictions.

### 2.5. Identifying Microbial Culture Filtrate Combinations

*T. asperellum* 10264, *B. subtilis* S4-4-10, and *M. anisopliae* 3.11962 were fermented separately in optimized media. The culture filtrates were mixed in different proportions (T:B:M = 1:1:1, 1:1:2, 1:2:1, 2:1:1, 1:1:3, 1:3:1, 3:1:1, 1:1:4, 1:4:1, 4:1:1, 1:2:2, 2:1:1, 2:2:1, 1:3:3:, 3:1:3, 3:3:1, 1:4:4, 4:1:4, and 4:4:1) and then mixed with PDA (1:3 (*V*/*V*)). The inhibitory effects of the culture filtrates mixed at different proportion levels against *R. solani* and *M. oryzae* were used as evaluation indicators to determine their optimal combination (TMB). This experiment was conducted with three biological replicates.

### 2.6. Determining the Characteristic Products of TMB

6-n-pentyl-2H-pyran-2-one (6-PP), alamethicin, and hydrophobin were measured using assay kits (ZY-65226, ZY-65296, ZY-65248; Zeye BioTechnology, Shanghai, China). β-Glucanase, gibberellin (GA), calmodulin (CaM), chitinase (Chitinase), indole-3-acetic acid (IAA), salicylic acid (SA), and cellulase (CE) were measured using the respective ELISA reagent kits (MM-9117201, MM-9156601, MM-92681401, MM-106201, MM-9156201, MM-9148601, and MM-9150201; Beijing Institute of Chemical Research, Beijing, China). Each treatment was repeated three times.

### 2.7. Observation of the Ultrastructure of C. suppressalis Larvae

Chopped fresh water bamboo (15 g) was mixed with 20 mL of TMB (200 rpm/min, 2 h) to fully absorb the metabolic solution. Then, the water bamboo was dried on sterile filter paper and placed in a culture dish (4 g) with 20 *C. suppressalis* larvae (3rd instar). The experiment was conducted with three biological replicates and included following the five treatments: distilled water (CK), single treatment with culture filtrate of *T. asperellum* 10264 (T), single treatment with culture filtrate of *B. subtilis* S4-4-10 (B), single treatment with culture filtrate of *M. anisopliae* 3.11962 (M), and mixed treatment with TMB (TMB = 1:4:1).

The midguts of *C. suppressalis* larvae, pre-treated with TMB for 24 h, were fixed with 2.5% glutaraldehyde (6 h) and 1% osmic acid (1.5 h), washed with 0.1 M PBS, and dehydrated with a graded ethanol series [22]. The samples were embedded in resin, and the surrounding area was polished until smooth. Slices with a thickness of less than 100 nm were cut and observed using JEM-1200EX TEM (JEOL, Tokyo, Japan).

### 2.8. RNA-Seq of C. suppressalis Larvae Treated with TMB

To explore the effect of TMB on the gene expression of *C. suppressalis* larvae, RNA-Seq analysis was carried out. Treated *C. suppressalis* larvae were frozen with liquid nitrogen for further study. Total RNA was extracted from the tissue using the TRIzol reagent. After the RNA quality was determined using the 2100 Bioanalyzer (Agilent, Santa Clara, CA, USA), mRNA with a poly (A) structure from the total RNA was enriched using oligonucleotide (dT) magnetic beads. Subsequently, cDNA was obtained through reverse transcription, and an RNA-Seq library was established, followed by sequencing on the Illumina NovaSeq 6000 sequencer (300 bp). Based on the gene expression level, differentially expressed genes (DEGs) were obtained among the groups. TBtools 2.121 was used to perform GO enrichment and KEGG enrichment on the DEGs.

### 2.9. RNA-Seq of M. oryzae Treated with TMB

Three cakes of *M. oryzae* (d = 5 mm) were inoculated into 250 mL conical flasks containing 100 mL of PD medium and T, M, B and TMB and cultured for 3 days. Collected mycelia were frozen with liquid nitrogen for RNA-Seq, with 3 replicates for each treatment. The analysis method was the same as that described above.

### 2.10. qRT-PCR Verification

To validate the RNA-Seq results, genes related to heat shock proteins, serine proteases, and transmembrane proteins were selected for qRT-PCR. The sample preparation and total RNA extraction methods were the same as those used for RNA-Seq. Each RNA sample was reversed using the HiScript III RT SuperMix for qPCR (Vazyme, Nanjing, China) to obtain cDNA. The qRT-PCR experiment was conducted using the ChamQ Universal SYBR qPCR Master Mix (Vazyme, Nanjing, China) with the Roche LightCycler 96 (Roche, Rotkreuz, Switzerland). The used primers are listed in Tables S6 and S7. *EF1* and *β-tubulin* were used as a stable internal reference gene of *C. suppressalis* and *M. oryzae* for relative quantification of the target genes. The expression levels of the target genes were calculated using the 2^−ΔΔCT^ method [23,24].

### 2.11. Determination of the Growth Rate of M. oryzae

Mycelia treated with different culture filtrate were collected, placed in a 50 °C oven for drying, and their dry weights were measured. Each treatment was carried out with three biological replicates.

### 2.12. Determination of the Gene Expression Related to Defense Response in Rice

Rice (Nangeng 46) seeds were soaked in clean water before being sown in field plots for the trial. The rice plants were sprayed with one of the five following treatments: (1) clean water; (2) culture filtrate of *T. asperellum* 10264; (3) culture filtrate of *B. subtilis* S4-4-10; (4) culture filtrate of *M. anisopliae* 3.11962; (5) mixed culture filtrates of TMB (*T. asperellum* 10264, *B. subtilis* S4-4-10, and *M. anisopliae* 3.11962 = 1:1:1). The culture filtrates were used at a dosage of 500 mL/666.7 square meters for each treatment area. After 3 days of treatment, rice leaf sheaths were collected at the 6-leaf stage from plants in the field for RNA extraction, followed by cDNA synthesis through reverse transcription. The primers used are listed in Table S8. Using *Ubi* as the internal reference gene, the relative expression levels of the target genes in rice under the different treatments were calculated using the 2^−ΔΔCT^ method [25].

### 2.13. Determination of the Enzyme Activity Related to Defense Response in Rice

TMB (2 mL) was diluted 10 times, and then 0.6 g of biochar and 0.3% sodium carboxymethyl cellulose were added to obtain the seed soaking agent. The rice seeds were disinfected with 75% alcohol on the surface for 5 min, and 0.4 g of rice seeds was germinated in 20 mL of seed soaking agent for two days. The rice seeds then were removed and left to germinate for 5 days. Subsequently, the well-germinated seeds were planted in a potted plant. The single culture filtrate and sterile water were the controls.

Rice leaves were collected at the six-leaf stage to measure the phenylalanine ammonia lyase (PAL), superoxide dismutase (SOD), and catalase (CAT) activity. Assay kits (BC0215, BC0175, and BC0205; Solarbio Science & Technology Co., Ltd., Beijing, China) were utilized to measure the PAL, SOD, and CAT activity, respectively.

### 2.14. Statistical Analysis

The experiments in this study were repeated at least three times, and Minitab 19 was used to analyze the data collected from the Plackett–Burman and CCD experiments. The significance of differences among the different treatments using the least significant difference (LSD) method. Tables and figures were processed using Microsoft Excel 2019, GraphPad Prism 9, Origin 2024b, and Adobe Illustrator 2020.

## 3. Results

### 3.1. Determination of the Optimal Culture Medium to Produce a Culture Filtrate with Highly Inhibitory Effects on Pathogens and Insect Larvae

Based on the inhibitory effects of the culture filtrate of each strain grown in the medium containing a single replaced nutritional component against *R. solani* and *M. oryzae*, maltose and galactose (carbon sources), skimmed milk powder (nitrogen sources), ammonium sulfate, magnesium sulfate heptahydrate, and potassium dihydrogen phosphate (inorganic salts) were selected for the PB design of the subsequent culture medium for *T. asperellum* 10264. Similarly, molasses and galactose (carbon sources), yeast powder and beef extract (nitrogen sources), and ammonium sulfate, magnesium sulfate heptahydrate, and potassium dihydrogen phosphate (inorganic salts) were selected for the subsequent PB test of *B. subtilis* S4-4-10. Two carbon sources (molasses and sucrose), two nitrogen sources (soybean peptone and beef extract), and four inorganic salts (ammonium sulfate, magnesium sulfate heptahydrate, potassium hydrogen phosphate, and potassium dihydrogen phosphate) were selected for the PB experiment of *M. anisopliae* 3.11962 (Figure S1).

The PB experiment was designed using factors selected through single-factor experiments. Significant influencing factors were identified based on the inhibitory effects of culture filtrates against *R. solani* and *M. oryzae*, and *C. suppressalis*. The results show that the culture filtrate of *T. asperellum* 10264 against *R. solani* and *C. suppressalis* did not have a significant inhibitory effect, while potassium dihydrogen phosphate, galactose, maltose, and ammonium sulfate significant influenced its inhibitory effect against *M. oryzae* (*p* < 0.01) (Table S9). Therefore, potassium dihydrogen phosphate, galactose, maltose, and ammonium sulfate were selected for the CCD, and the inhibitory rate against *M. oryzae* was selected as the response value for *T. asperellum* 10264. In contrast, the three response values for the *B. subtilis* S4-4-10 culture filtrate did not exceed the three significant influencing factors (Table S10), although their inhibition rates reached over 85%. Further optimization of the culture medium of *B. subtilis* S4-4-10 was not carried out. In the PB test for the *M. anisopliae* 3.11962 culture medium, the response value to *R. solani* was not significant. Taking into account the inhibition rate of *M. oryzae* and the mortality rate of *C. suppressalis* (*p* < 0.05) (Table S11), ammonium sulfate, molasses, soy peptone, and magnesium sulfate were selected for subsequent experiments.

The center points and step sizes of the CCD of *T. asperellum* 10264 and *M. anisopliae* 3.11962 were determined through the steepest climbing test (Table S12). The central point concentrations for the CCD of *T. asperellum* 10264 were as follows: 0.8 g of dipotassium hydrogen phosphate, 24 g of galactose, 16 g of maltose, and 1.2 g of ammonium sulfate. The central point concentrations for the CCD of *M. anisopliae* were as follows: 0.7 g of ammonium sulfate, 24 g of molasses, 6.8 g of soy peptone, and 0.9 g of magnesium sulfate.

**Figure 1:**
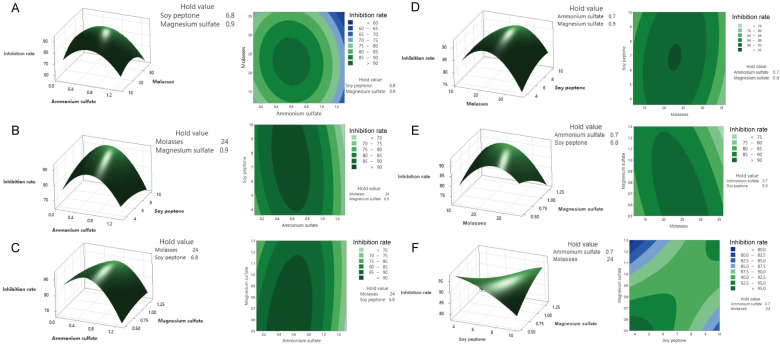
Response surface diagrams and contour maps of the central composite designs of the culture medium components for *M. anisopliae* 3.11962 metabolites’ inhibitory activity against *M. orizae*. (**A**) The interaction between the inhibition rate and the concentrations of ammonium sulfate and molasses (left: curve plot; right: contour map; the same applies below); (**B**) the interaction between the inhibition rate and the concentrations of ammonium sulfate and soybean peptone; (**C**) the interaction between the inhibition rate and the concentrations of ammonium sulfate and magnesium sulfate; (**D**) the interaction between the inhibition rate and the concentrations of molasses and soybean peptone; (**E**) the interaction between the inhibition rate and the concentrations of molasses and magnesium sulfate; (**F**) the interaction between the inhibition rate and soybean peptone and magnesium sulfate.

In the CCD experiment, the response interactions among the four important variables of potassium dihydrogen phosphate, galactose, maltose, and ammonium sulfate in the culture medium of *T. asperellum* 10264 were clarified, and the following fitting equation was obtained: Y2 (*M. oryzae*%) = 59.535 − 2.305 A − 0.473 B + 0.368 C + 4.985 D − 1.080 A × A + 1.030 C × C + 2.086 A × D + 1.804 B × D − 1.154 C × D. The regression coefficients of determination for the model were R-seq = 99.07%, R-seq (Adj) = 98.51%, and R-seq (predicted) = 97.45%, indicating a good fit between the model and the actual situation. Based on the obtained experimental analysis of variance results and quadratic regression equations, response surface analysis and contour maps for the different factors are shown in Figure S2. Further observation of the interaction between factors revealed that the interactions among potassium dihydrogen phosphate and galactose had the most significant impact. Similarly, the response interactions among the four important variables of ammonium sulfate, molasses, soy peptone, and magnesium sulfate in the culture medium of *M. anisopliae* 3.11962 were clarified, and the following regression equation was fitted: Y2 = 92.075 − 2.716 A − 1.004 B + 0.167 C − 1.022 D − 3.867 A × A − 2.351 B × B−0.545 C × C − 0.439 D × D − 0.805 A × C + 0.908 A × D − 1.234 B × D + 1.801 C × D. The regression coefficient of determination for the model was R-seq = 96.96%, R-seq (Adj) = 94.94%, and R-seq (prediction) = 89.39% (Figure 1).

### 3.2. Determination of Biomarkers for Culture Filtrates

We conducted biomarker assays on the T, M, B, and TMB treatments. The results show that the salicylic acid (SA) content was highest in the T treatment. The contents of 6-pentyl-2-pyranone (6PP), auxin (IAA), and cellulase were the highest in the M treatment. In treatment B, the contents of alamethicin, hydrophobin, gibberellin, chitinase, and glucanase were the highest. The contents of hydrophobin and calmodulin in the TMB treatment were lower than those in all single microbial metabolic fluids, while the contents of the other indicators were at a moderate level. Moreover, the content of free amino acids in TMB was 99.263 μg/mL, while the contents of free amino acids in T, M, and B were 17.478 μg/mL, 326.422 μg/mL, and 347.566 μg/mL, respectively (Table S13). Although the content of free amino acids in TMB was lower than that of the highest single metabolite, its variety was relatively more comprehensive, which may be the reason for its best antifungal and insecticidal performance.

### 3.3. Antifungal and Insecticidal Effects of TMB

**Figure 2:**
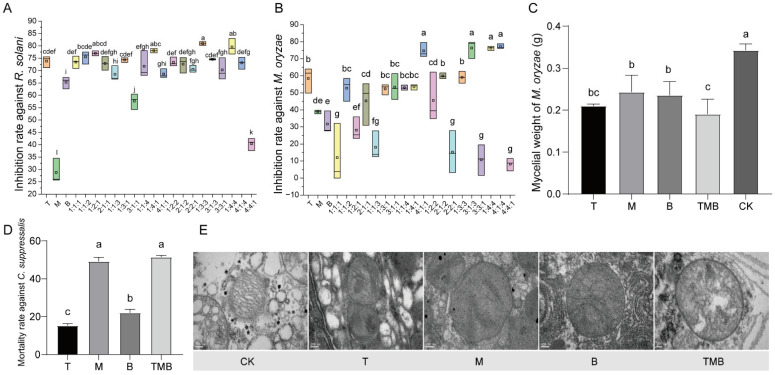
The inhibition effects of TMB against *R. solani*, *M. oryzae*, and *C. suppressalis*. (**A**) The inhibition rate against *R. solani*. (**B**) The inhibition rate against *M. oryzae*. (**C**) The effect of metabolic fluids on the growth of *M. oryzae*. (**D**) The mortality rate of *C. suppressalis*. (**E**) The ultrastructure of *C. suppressalis* under a transmission electron microscope (TEM). Bars = 200 nm (CK, T, M, and B) and 100 nm (TMB). CK (control): distilled water treatment; T: single treatment with culture filtrate of *T. asperellum* 10264; B: single treatment with culture filtrate of *B. subtilis* S4-4-10; M: single treatment with culture filtrate of *M. anisopliae* 3.11962; TMB: mixed treatment with TMB (1:4:1). Note: Different lowercase letters represent significant differences between groups (*p* < 0.05).

As depicted in Figure 2A,B, the single culture filtrates of *T. asperellum* 10264 and *B. subtilis* S4-4-10 did not have ideal inhibitory effects against *R. solani* and *M. oryzae*. However, some mixed proportions of the strain culture filtrates revealed higher antifungal effects than those of the single culture filtrates. The inhibitory rate of TMB (=1:4:4) against *R. solani* reached 82.47%, which was significantly higher than those of the culture filtrates of *T. asperellum* 10264 at 73.83%, *M. anisopliae* 3.11962 at 28.76%, and *B. subtilis* S4-4-10 at 65.29%. The inhibitory rate of TMB (=1:4:4) against *M. oryzae* was significantly higher than that of the single culture filtrates of *T. asperellum* 10264 and *B. subtilis* S4-4-10. Compared with the control, the culture filtrate significantly inhibited the growth of *M. oryzae*. Among them, TMB generated the most significant inhibitory effects against pathogens (Figure 2C).

As shown in Figure 2D, when the mixed ratio of the three culture filtrates (TMB) was 1:4:1, the mortality rate of the mixed filtrate on *C. suppressalis* was 51%, which was higher than those of the single culture filtrates. Additionally, both the single and compound culture filtrate treatments were able to significantly change the mitochondrial structure of the *C. suppressalis* larva (Figure 2E), with the effect of the TMB treatment being the most significant, resulting in severe spinal structure rupture. It can be speculated that culture filtrates can damage the mitochondrial structure of *C. suppressalis* larva, thereby affecting larva respiration. In addition, it was found that the culture filtrate of *M. anisopliae* 3.11962 caused deformation of the Golgi apparatus.

### 3.4. RNA-Seq Analysis of C. suppressalis Larvae After TMB Treatment

The reads were filtered following transcriptome sequencing, yielding 96.73 Gb of clean data, with the percentage of Q30 bases being over 94% (Table S14). A total of 15,653 expressed genes were detected, all representing known genes. Twelve genes related to heat shock protein and serine protease were selected for validation. The changes in the 12 genes as determined by qRT-PCR were consistent with those of the RNA-Seq analyses (Figure S3), indicating that our RNA-Seq data were reliable.

**Figure 3:**
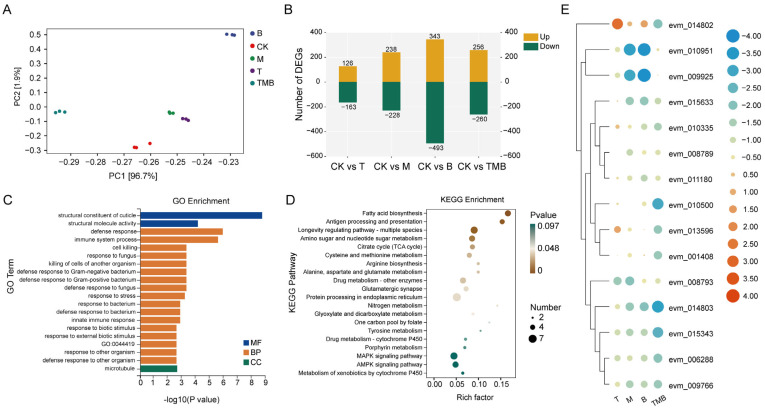
The effects of TMB on the gene expression of *C. suppressalis*. (**A**) PCA analysis of DEGs. (**B**) The number of DEGs after treatment with different metabolites. (**C**) GO enrichment analysis of the TMB treatment. GO:0044419 is the biological process involved in interspecies interactions between organisms. (**D**) KEGG enrichment analysis of the TMB treatment. (E) The main DEGs related to immunity in *C. suppressalis* exposed to treatment compared to the untreated groups (Table S15).

The differential expression genes (DEGs) of *C. suppressalis* larvae showed significant variations among the treatments of different culture filtrate (Figure 3C). Compared with the control, 516 DEGs were found in the TMB treatment, among which 256 were upregulated and 260 were downregulated (Figure 3B).

The DEGs were annotated to the Gene Ontology (GO) database, and the top 20 ranked GO terms (*p* <0.05) are listed in Figure 3C and Figure S4A–C. The GO enrichment results showed that the T treatment mainly caused “response to fungus”, “cell killing”, and “defense response to fungus”, among other responses. The M and B treatments mainly induced changes in gene expressions related to chitin synthesis and amino acid and carbohydrate metabolism, such as “chitinase activity”, “amino sugar metabolic process”, “carbohydrate metabolic process”, and “aminoglycan metabolic process”, among others. Among the terms in TMB, the most significant enrichment terms were “structural constituent of cuticle”, “defense response”, “immune process”, and other related terms.

Compared with the control group, the DEGs associated with the treatment group were annotated to the Kyoto Encyclopedia of Genes and Genomes (KEGG) database. The highly KEGG-enriched pathways in the T treatment included “caffeine metabolism”, “drug metabolism—other enzymes”, “valine, leucine and isoleucine biosynthesis”, and “oxidative phosphorylation”. The DEGs related to “amino sugar and nucleotide sugar metabolism”, “fatty acid biosynthesis”, and “AMPK signaling pathway” were changed in M. Finally, the DEGs related to life cycles, such as “amino sugar and nucleotide sugar metabolism”, “DNA replication”, and “cell cycle”, were affected by B. The main metabolic pathways affected by TMB included “fatty acid biosynthesis”, “amino sugar and nucleotide sugar metabolism”, “citrate cycle (TCA cycle)”, and the “MAPK signaling pathway” (Figure 3D and Figure S4D–F).

We also observed that GO terms related to the immune system of *C. suppressalis* were significantly affected. A total of 15 immune-related DEGs were subsequently analyzed using cluster analysis (Figure 3E). The results show that all culture filtrate treatments could significantly change the expression levels of immune-related genes, with the most significant downregulation of DEGs observed with TMB treatment. Compared to the control, DEGs encoding chitinase (evm_010951, evm_015633, evm_008789, evm_008793, and evm_009925) were downregulated by the M treatment. However, TMB and T downregulated only one chitinase related gene each, which were “evm_015633” and “evm_008793”, respectively. All treatments downregulated the Attacin-A encoding genes (evm_014802, evm_015343, and evm_009766), but DEGs encoding growth arrest and DNA damage-inducible protein GADD45 alpha (evm_010335), ATP-dependent DNA helicase PIF1 (evm_010500), and N-glycosylase/DNA lyase protein GADD45 alpha (evm_001408), were downregulated only by TMB. These genes were also related to the growth and development of *C. suppressalis*, which is consistent with the TEM results, indicating that they may be potential targets of TMB.

### 3.5. RNA-Seq Analysis of M. oryzae Treated with TMB

**Figure 4:**
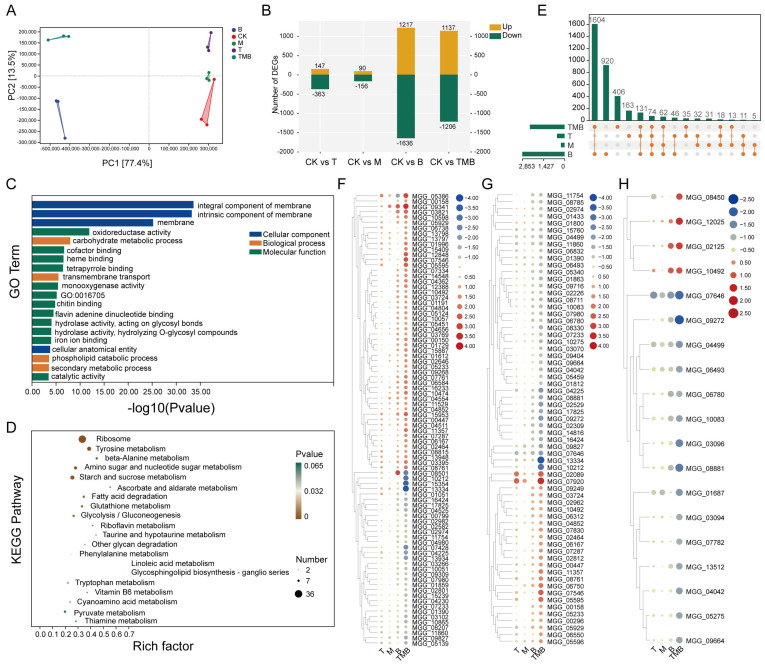
The effects of TMB on *M. oryzae*. (**A**) The number of DEGs after treatment with different metabolites. (**B**) PCA analysis of DEGs of *M. oryzae*. (**C**) The effects of culture filtrates on the growth of *M. oryzae*. (**D**) GO enrichment analysis. (**E**) KEGG enrichment analysis. (**F**) The main DEGs related to membrane in *M. oryzae* exposed to treatment compared to untreated groups (Table S17). (**G**) Major DEGs related to protein/transcription factors in *M. oryzae* exposed to treatment compared to untreated groups (Table S18). (**H**) Major DEGs related to metabolic processes in *M. oryzae* exposed to treatment compared to untreated groups (Table S19).

The reads were filtered following transcriptome sequencing, yielding 102.76 Gb of clean data, with the percentage of Q30 bases being over 98% (Table S16). A total of 12,825 expressed genes were detected, all representing known genes. Principal component analysis (PCA) showed that the treatment and control groups were well separated, indicating that the gene expression of *M. oryzae* was significantly affected by the different culture filtrates (Figure 4A). Compared with the control, the number of DEGs caused by B was the highest (2853 DEGs), while the number of DEGs caused by T and M were 510 and 246, respectively. The number of DEGs caused by TMB was between those of the B, T and M treatments, with totals of 1137 upregulated and 1206 downregulated genes (Figure 4B).

To verify the expressions of DEGs in RNA-Seq, we randomly selected eight genes to undergo qRT-PCR to estimate their expression levels. The changes in the eight genes as determined through qRT-PCR were consistent with those of the RNA-Seq analyses (Figure S5), revealing that our RNA-Seq data were reliable.

GO enrichment analysis found that all treatment groups caused changes in the membrane composition of *M. oryzae*, but the TMB and B treatments caused the most DEGs, with 480 and 573 DEGs, respectively. T led to more changes in transport protein pathways (GO:0022804, GO:0015291, GO:0055085, etc.). Meanwhile, M caused more changes in amino acid-related catabolic processes (GO:1901606, GO:0009063, GO:0009074, etc.). B caused changes in ribosome-related terms (GO:0005840, GO:0003735, GO:0044391, etc.), while TMB caused more changes in binding activity and metabolic processes (GO:0020037, GO:0046906, GO:0005975, etc.) (Figure 4C and Figure S6A–C).

According to KEGG enrichment analysis, T mainly affected the expressions of genes related to pathways such as “methane metabolism”, “glycine, serine and threonine metabolism”, and “phenylalanine metabolism”. M affected the expression of genes related to “tyrosine metabolism”, “phenylalanine metabolism”, “ubiquinone and other terpenoid-quinone biosynthesis”, etc. Meanwhile, B mainly interfered with genes related to “ribosome”, “base excision repair”, “beta-Alanine metabolism”, and so on. Among these, the pathway genes related to ribosomes were the most significantly affected. The highly enriched pathways in TMB included “ribosome”, “tyrosine metabolism”, and “beta-Alanine metabolism” (Figure 4D and Figure S6D–F).

We found that TMB resulted in 406 unique DEGs (Figure 4E). Subsequently, we conducted GO enrichment analysis on these DGEs separately and found that they were mainly enriched in membrane-related functions, protein/transcription factors, and metabolic processes (Figure S7).

Among the 406 DEGs, 86 were enriched in membrane component-related terms. Cluster analysis showed that compared with other treatments, DEGs such as MGG_08501, MGG_10212, and MGG_15354 were significantly downregulated under the TMB treatment, while MGG_07428 and MGG_04225 were upregulated (Figure 4F). In the protein/transcription factor related pathway, DEGs such as MGG_13334 and MGG_10212 were significantly downregulated, and MGG_02089 and MGG_07920 were significantly upregulated under the TMB treatment (Figure 4G). DEGs such as MGG_08450, MGG_12025, etc. were significantly upregulated, while DEGS (MGG_07646, MGG_09272, etc.) were significantly downregulated in metabolic processes (Figure 4H). The above results indicate that TMB might inhibit the growth and development of *M. oryzae* by disrupting its cell membrane permeability and protein metabolism.

### 3.6. Effects of TMB on Rice Defense Response

**Figure 5:**
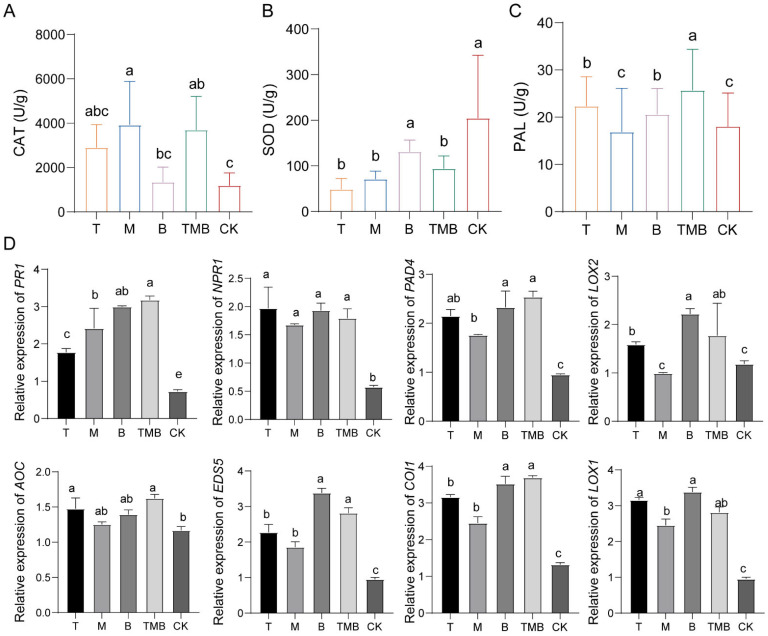
The effects of TMB on enzyme activity and defense genes in rice. (**A**) CAT enzyme activity. (**B**) SOD enzyme activity. (**C**) PAL enzyme activity. (**D**) The expressions of genes related to rice defense response signals. Note: Different lowercase letters represent significant differences between groups (*p* < 0.05).

The defense-related enzyme activity of seedlings grown from rice seeds soaked in culture filtrates was increased under T, B, M, and TMB treatments. The highest catalase (CAT) activity was obtained with M, the highest superoxide dismutase (SOD) activity was obtained with B, and the highest phenylalaninase (PAL) activity was obtained with T (Figure 5A–C). Soaking the seeds in TMB induced activities of CAT and PAL up to the highest level, reaching 3692.54 ± 1513.15 U/g and 25.69 ± 2.08 U/g, respectively, which were significantly higher than those of seeds soaked in a single culture filtrate. The SOD activity was the same as that mentioned above.

Different culture filtrates induced different expression levels of defense response-related genes in rice. This trend was observed at different time points (Figure 5D). The *NPR1*, *PR1*, and *PAD4* genes of the salicylic acid (SA) signaling pathway were significantly upregulated under all treatments. Overall, their upregulation was more significant after treatment with TMB, which indicates that TMB causes significant expression of the SA signaling pathway. The signaling pathways of jasmonic acid (JA) and ethylene (ET) were also measured, and *LOX1*, *LOX2*, and *AOC* showed an upregulation trend. However, their upregulation under the TMB treatment was not the most significant.

## 4. Discussion

In the past few years, some studies have reported on the use of *Trichoderma* spp., *Metarhizium* spp., and *Bacillus* spp. and their culture filtrates alone for the prevention and control of crop diseases and pests [26,27,28,29,30,31]. However, the effect of a single strain filtrate for controlling crop diseases is limited. To date, there have been no reports on the development of combined culture filtrates of three microbes or on understanding its biocontrol mechanism against pathogens and pest insects in rice.

Our research is the first report to develop a new kind of combined culture filtrate agent and determine its action mechanism against sheath blight, blast, and stem insects in rice. The combined culture filtrate agent, TMB, was identified as the most effective new biocontrol agent, providing a new perspective for the green prevention and control of rice pests and diseases. This study developed a preparation method for the composite culture filtrate agent (TMB) by selecting the optimal culture medium and mix ratio. The optimized agent exhibited significantly better antifungal and insecticidal activities against *R. solani*, *M. oryzae*, and *C. suppressalis*, as well as ISR activities in rice compared to a single culture filtrate, indicating the advantage of compound culture filtrates sourced from different isolates of biocontrol microbes over a single strain culture filtrate.

The mode of action of the tested culture filtrates against *M. oryzae*, *R, solani*, and *C. suppressalis* may be due to the inhibition of pathogen or pest insect gene expression as well as the destruction of plant tissues or pest insect midgut ultrastructure. It was suggested that TMB may kill the larvae of *C. suppressalis* by disrupting their midgut ultrastructure and interfering with their insect immune response system. Previous work has demonstrated that *M. anisopliae* can cause severe damage to the midgut epithelial cells and salivary glands of *Spodoptera litura* larvae [32], as well as ultrastructure changes to the midgut epithelial cells and villi in the lepidopteran pest *Galleria Melonella* [33], by producing the cyclic peptide fungal toxin destruxin. However, no work has demonstrated the direct action of microbial culture filtrates against pest insects, particularly, the function of combined culture filtrates from different isolates of biocontrol microbes against larvae of *C. suppressalis*. Our study found that the insect midgut mitochondrial cristae and Golgi apparatus of *C. suppressalis* larvae were ruptured and deformed after the TMB treatment, suggesting that TMB may interfere with energy metabolism, nutrient transportation, and the immune system by reducing the synthesis of sugars, proteins and lipids, as well as impairing signal transduction. Insects have a powerful and effective immune system that enables them to resist microbial invasion. Several antimicrobial peptides have been isolated and classified in insects, such as attacin, cecropin, defencin, gallerimycin, and lysozyme, which are regarded as a self-defensive mechanism to resist stress conditions and biocontrol [34,35]. For instance, when insects are infected by bacteria or fungi, they may produce antimicrobial peptides to prevent the proliferation of aggressive microbes in the body or directly kill them. Our results suggest that combined culture filtrates may not directly kill pest insects, but can suppress their immune response, thus making them more sensitive to the culture filtrate. Similarly, it was found that genes related to Attacin-A (evm_014802, evm_015343, and evm_009766) in *C. suppressalis* larvae were all downregulated by the culture filtrates, especially TMB. Therefore, Attacin-A may be involved in insect immune resistance to pathogen infection. Shahriari et al. observed that the gene expression levels of AMPs (i.e., attacin 1 and 2, cecropin 1 and 2, defencin, and gallerimycin) measured after fungal injection were increased in a short time, thereby increasing insect resistance against pathogen invasion [36]. Moreover, the gene expressions of defense protein (evm_014803) and cecropin (evm_013596) in insects were also significantly downregulated by the TMB treatment. In addition, it was found that the expression levels of growth arrest and DNA damage-inducible protein (GADD45)(evm_010335) in *C. suppressalis* were significantly downregulated after TMB treatment, since GADD45 is a member of the gene family associated with cell growth regulation, apoptosis, and DNA damage repair [37]. Similarly, Peretz et al. [38] found that the GADD45 transcript was increased once the animal immune response was activated. ATP-dependent DNA helicase PIF1 (evm_010500) and N-glycosylase/DNA lyase (evm_001408) related to DNA repair were significantly downregulated [39,40] if the insect immune system was impaired. The above results indicate that culture filtrate, especially TMB, can kill insects mainly by interfering with their immune systems.

The transcriptome analysis revealed that disruption of the membrane structure decreased the activity of transport proteins/transcription factors and reduced energy metabolism. These are proposed as the main potential mechanisms by which culture filtrates inhibit pathogens, similar to chemical fungicides that can exert antifungal effects by damaging fungal cell membranes [41,42,43]. Li W et al. [44] verified that tetramycin can destroy cell membrane integrity, resulting in the leakage of cellular components such as nucleic acids and proteins in mycelial suspensions. The DAL5 gene encodes a component of the uric acid transport system, which is closely related to the function of intact membrane proteins [45]. It was found that TMB could inhibit rice blast pathogens by downregulating allantoate permease (MGG_07428), which is related to membrane and transport systems in *M. oryzae*, leading to the destruction of rice blast fungus cell membrane integrity. TMB can also decrease pathogen tolerance to environmental stress conditions by downregulating a heavy metal tolerance protein (MGG_11754), indicating that the membrane transport system of *M. oryzae* loses functionality after culture filtrate treatment. In our study, AGP2 (MGG_13334), sugar transporter family protein (MGG_09827), metabolite transporter (MGG_07980), and MFS hexose transporter (MGG_01390) were significantly downregulated because general amino acid permease AGP2 responds to environmental cues and controls the expression of several transporter genes, including several hexose, nucleoside, and vitamin permease genes [46]. Disruption of pathogens’ metabolic processes is a crucial mechanism by which TMB inhibits pathogens. It was found that most of the DEGs in the carbohydrate metabolism, organic acid metabolism, and metabolic processes in *M. oryzae* were downregulated after the TMB treatment. Genes such as β-glucanase (MGG_06493) and α-glucuronidase (MGG_07646) in carbohydrate metabolismwere significantly downregulated, indicating that TMB treatment may interfere with the homeostasis of *M. oryzae* cell walls and the process of plant infection. A similar work demonstrated that recombinant javanicin interfered with carbohydrate metabolism and energy production, affecting the glycolysis pathway and mitochondrial respiration of *Cryptococcus neoformans* [47]. Furthermore, the TMB treatment inhibited the gene expressions of glutamyl-tRNA synthetase, leucyl-tRNA synthetase, and aspartyl-tRNA synthetase in *M. oryzae*, thus hindering the synthesis of protein, carboxylic acid, and organic acid, which was found to be consistent with a previous work [48].

TMB may help to improve the immunity of rice by inducing defense response-related genes and enzyme activity expression to resist external pests and diseases. Recently, there have even been many studies indicating that microorganisms can induce plant immunity by secreting proteins or secondary metabolites. Wang et al. [49] purified a protein from *Bacillus amyloliquefaciens* that can induce the accumulation of ROS and phenolic compounds in tobacco and activate the upregulated expression of the salicylic acid pathway resistance marker genes *PR1* and *PR5*, as well as the jasmonic acid pathway resistance marker gene *pdf1.2*. Similarly, Yu et al. [50] found that the co-expression of class II hydrophobic protein Hyd1 (Thyd1) from *Trichoderma harzianum* and UBL (ubl) from maize synergistically promoted plant resistance to gray mold in *Arabidopsis*. ROS is an intracellular metabolite that plays an important role as a secondary messenger in many signaling pathways. However, excessive ROS can cause enzyme inactivation and membrane damage, even leading to plant cell death [51]. Our study has proven that microbial culture filtrate can stimulate increase in SOD, CAT, and PAL enzyme activity in rice, thereby helping to eliminate excessive ROS in stressed plants and preventing adverse external interference with them. From the perspective of biocontrolling plant diseases, TMB induced the expressions of defense response-related genes in rice, such as *NPR1*, *PR1*, and *PAD4* in the SA pathway, as well as *LOX1*, *LOX2*, *DES5*, *Col1*, and *AOC* in the JA and ET pathways, which were significantly upregulated. These results are consistent with previous research findings. The phytohormones SA and JA are major players in plant immunity that can help defend plants against pathogens [52]. Joglekar S et al. [53] found that thaxtomin A induced the expressions of several *EDS1*/*PAD4*-regulated genes, including *EDS1*, *PAD4*, and *PR1*, promoting the accumulation of SA.

## 5. Conclusions

In summary, we successfully developed a new composite culture filtrate agent, TMB, by combining three biocontrol microorganisms (*T. asperellum* 10264, *B. subtilis* S4-4-10, and *M. anisopliae* 3.11962) in an optimized model. The TMB demonstrated high inhibitory effects against *R. solani*, *M. oryzae*, and *C. suppressalis* larvae and was able to induce immune responses in rice. The results of RNA-Seq analysis further suggested that TMB may kill the larvae of *C. suppressalis* by disrupting the mitochondria in the insect midgut. It may also inhibit rice blast fungus growth by disrupting the pathogen cell membrane structure, inhibiting the activity of transporters/transcription factors related to growth metabolism. Furthermore, TMB may induce immune responses in rice against rice disease and pest insect infection by triggering the SA and JA/ET signaling pathways. In conclusion, this study developed and demonstrated TMB as a new potential biocontrol agent for use in the control of rice pests and diseases, which could also be considered as a potential bio-stimulant consortium with multiple functions. In the future, TMB may be used in combination with chemical pesticides to help synergistically control crop diseases.

## Data Availability

The original contributions presented in this study are included in the article/Supplementary Materials. Further inquiries can be directed to the corresponding author.

## Supplementary Materials

The following supporting information can be downloaded at: https://www.mdpi.com/article/10.3390/jof11070471/s1, Figure S1 The inhibitory effects of metabolites prepared from different carbon sources, nitrogen sources, and inorganic salt culture media against *Rhizoctonia solani* and *Magnaporthe orizae*. Figure S2 Response surface diagram and contour map of the central composite design of the culture medium components for *T. asperellum* 10264 metabolites inhibitory activity against *M. orizae*. Figure S3 Validation of RNA-Seq results of *C. suppressalis* using qRT-PCR. Figure S4 GO enrichment analysis and KEGG enrichment analysis of DEGs in *C. suppressalis*. Figure S5 Validation of RNA-Seq results of *M. oryzae* using qRT-PCR. Figure S6 GO enrichment analysis and KEGG enrichment analysis of DEGs in *M. oryzae*. Figure S7 GO analysis of unique DEGs in TMB. Table S1 PB design for *Trichoderma asperellum* 10264 medium components. Table S2 PB design for *Bacillus subtilis* S4-4-10 medium components. Table S3 PB design for *Metarhizoma anisopliae* 3.11962 medium components. Table S4 Center combination design for *Trichoderma asperellum* 10264 medium components. Table S5 Center combination design for *Metarhizoma anisopliae* 3.11962 medium components Table S6 Primers of *C. suppressalis* larva used for qRT-PCR analysis Table S7 Primers of *M. oryzae* used for qRT-PCR analysis Table S8 Primers of Rice used for qRT-PCR analysis Table S9 Evaluation of the effect of each factor in the Plackett-Burman design of medium components for *T. asperellum* 10264. Table S10 Evaluation of the effect of each factor in the Plackett-Burman design of medium components for *B. subtilis* S4-4-10. Table S11 Evaluation of the effect of each factor in the Plackett-Burman design of medium components for *M. anisopliae* 3.11962. Table S12 The steepest climbing test design and results of *T. asperellum* 10264 and *M. anisopliae* 3.11962. Table S13 Types and contents of biomarkers in different culture filtrates. Table S14 Summary of RNA-seq reads of *C. suppressalis* treated in different metabolites. Table S15 Summary table of major DEGs related to immunity in *C. suppressalis* exposed to treatment compared to untreated groups. Table S16 Summary of RNA-seq reads of *M. oryzae* treated in different metabolites. Table S17 Summary of major DEGs related to membrane in *M. oryzae* exposed to treatment compared to untreated group. Table S18 Summary of major DEGs related to protein/transcription factor in *M. oryzae* exposed to treatment compared to untreated groups. Table S19 Summary of major DEGs related to metabolic process in *M. oryzae* exposed to treatment compared to untreated groups.
