# Supplementary material for: Optimized Combinations of Filtrates of Trichoderma spp., Metarhizium spp., and Bacillus spp. in the Biocontrol of Rice Pests and Diseases
Source: J Fungi (Basel). 2025 Jun 20;11(7):471. doi: 10.3390/jof11070471 (PMC12295696; doi:10.3390/jof11070471)
Supplement: Supplementary file 1 [file jof-11-00471-s001.zip › jof-3655884-supplementary.pdf]

The role of optimized combinations of cultural filtrates of certain microbial isolates in the biocontrol of rice pests and diseases

Xifen Zhang[#12], Lusheng Chen[#12], Zhenxu Bai[12], Yaqian Li[12], Jie Chen*[12]

School of Agriculture and Biology, Shanghai Jiao Tong University

State Key laboratory of microbial metabolism, Shanghai Jiao Tong University

[#] Xifen Zhang and Lusheng Chen contributed equally to this work. Author order was determined on the basis of their contributions for this paper.

* Corresponding author: jiechen59@sjtu.edu.cn

Captions

Figure S1 The inhibitory effects of metabolites prepared from different carbon sources, nitrogen sources, and inorganic salt culture media against *Rhizoctonia solani* and *Magnaporthe orizae*. 4

Figure S2 Response surface diagram and contour map of the central composite design of the culture medium components for *T. asperellum* 10264 metabolites inhibitory activity against *M. orizae*........ 5

Figure S3 Validation of RNA-Seq results of *C. suppressalis* using qRT-PCR........ 6

Figure S4 GO enrichment analysis and KEGG enrichment analysis of DEGs in *C. suppressalis*. 7

Figure S5 Validation of RNA-Seq results of *M. oryzae* using qRT-PCR. 8

Figure S6 GO enrichment analysis and KEGG enrichment analysis of DEGs in M. oryzae.... 9

Figure S7 GO analysis of unique DEGs in TMB. 10

Table S1 PB design for *Trichoderma asperellum 10264* medium components. 11

Table S2 PB design for *Bacillus subtilis* S4-4-10 medium components. 12

Table S3 PB design for *Metarhizoma anisopliae* 3.11962 medium components........ 13

Table S4 Center combination design for *Trichoderma* asperellum 10264 medium components. 14

Table S5 Center combination design for Metarhizoma *anisopliae* 3.11962 medium components........ 15

Table S6 Primers of *C. suppressalis* larva used for qRT-PCR analysis........ 16

Table S7 Primers of *M. oryzae* used for qRT-PCR analysis 17

Table S8 Primers of Rice used for qRT-PCR analysis........ 18

Table S9 Evaluation of the effect of each factor in the Plackett-Burman design of medium components for *T. asperellum* 10264 19

Table S10 Evaluation of the effect of each factor in the Plackett-Burman design of medium components for *B. subtilis* S4-4-10 21

Table S11 Evaluation of the effect of each factor in the Plackett-Burman design of medium components for *M. anisopliae* 3.11962 23

Table S12 The steepest climbing test design and results of *T. asperellum* 10264 and *M. anisopliae* 3.11962. 25

Table S13 Types and contents of biomarkers in different culture filtrates........ 26

Table S14 Summary of RNA-seq reads of *C. suppressalis* treated in different metabolites... 27

Table S15 Summary table of major DEGs related to immunity in *C. suppressalis* exposed to treatment compared to untreated groups. 28

Table S16 Summary of RNA-seq reads of *M. oryzae* treated in different metabolites. 29

Table S17 Summary of major DEGs related to membrane in *M. oryzae* exposed to treatment compared to untreated groups 30

Table S18 Summary of major DEGs related to protein/ transcription factor in *M. oryzae* exposed to treatment compared to untreated groups 34

Table S19 Summary of major DEGs related to metabolic process in *M. oryzae* exposed to treatment compared to untreated groups 37

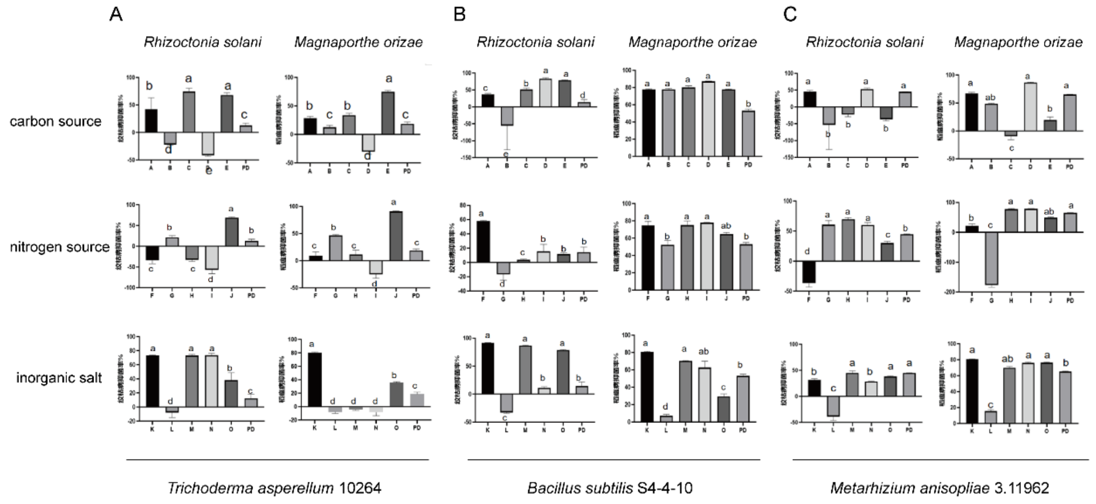

Figure S1 The inhibitory effects of metabolites prepared from different carbon sources, nitrogen sources, and inorganic salt culture media against *Rhizoctonia solani* and *Magnaporthe orizae*.

(A) The inhibition effect of T. asperellum 10264 metabolic fluids. (B) The inhibition effect of B. subtilis S4-4-10 metabolic fermentation. (C) The inhibition effect of M. anisopliae 3.11962 metabolic fermentation. Values with different letters are statistically significantly different ($P < 0.05$).

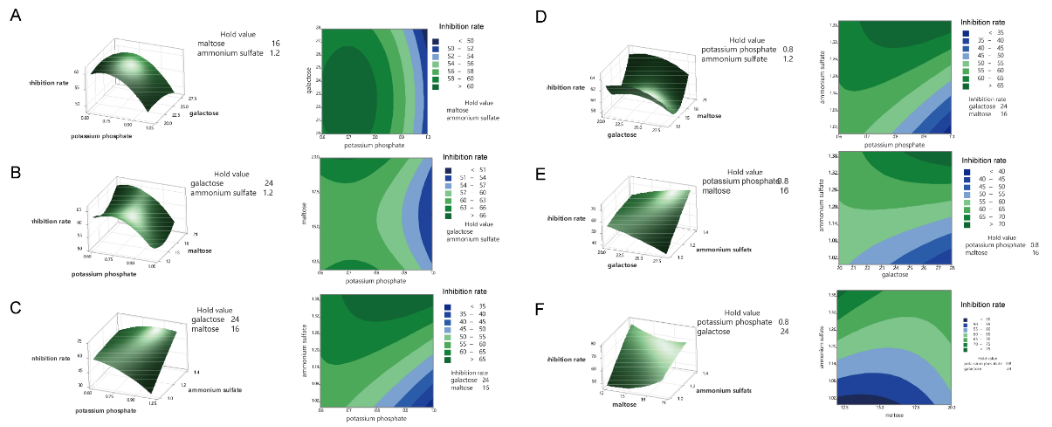

Figure S2 Response surface diagram and contour map of the central composite design of the culture medium components for *T. asperellum* 10264 metabolites inhibitory activity against *M. orizae*.

A: the interaction between the inhibition rate and dipotassium hydrogen phosphate and galactose (left: curve plot; right: contour map. The same below); B: the interaction between the inhibition rate and dipotassium hydrogen phosphate and maltose; C: the interaction between the inhibition rate and dipotassium hydrogen phosphate and ammonium sulfate; D: the interaction between the inhibition rate and maltose and galactose; E: the interaction between the inhibition rate and maltose and ammonium sulfate; F: the interaction between the inhibition rate and maltose and ammonium sulfate.

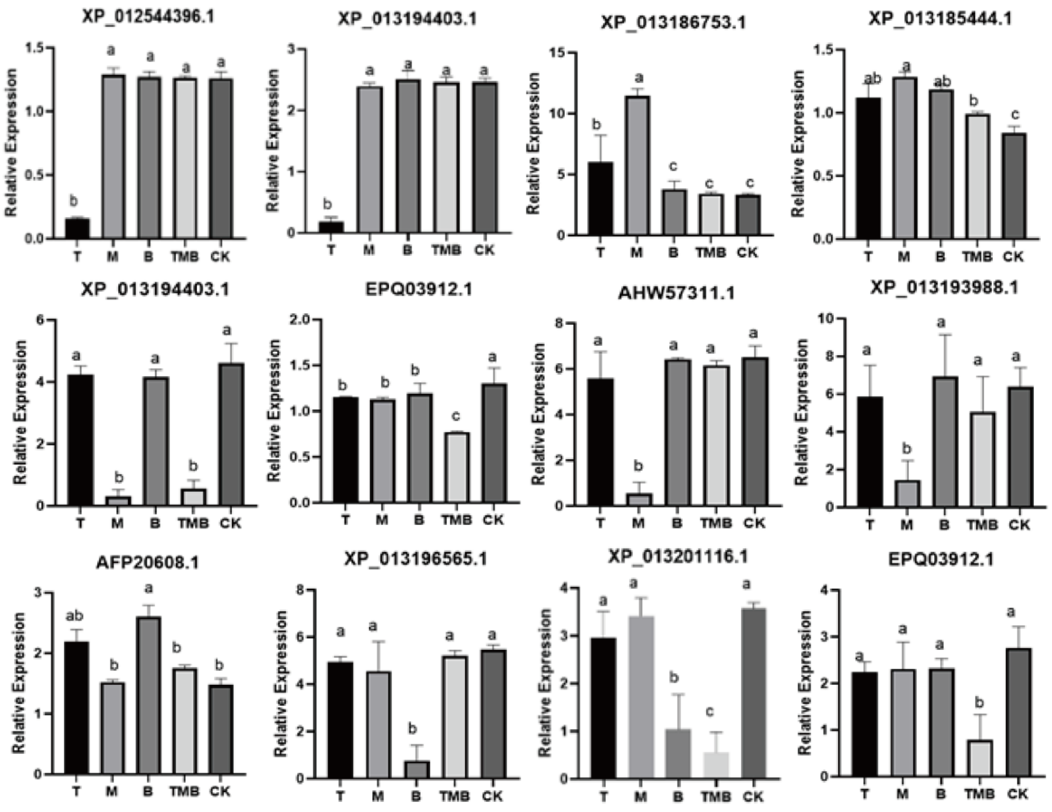

Figure S3 Validation of RNA-Seq results of *C. suppressalis* using qRT-PCR.

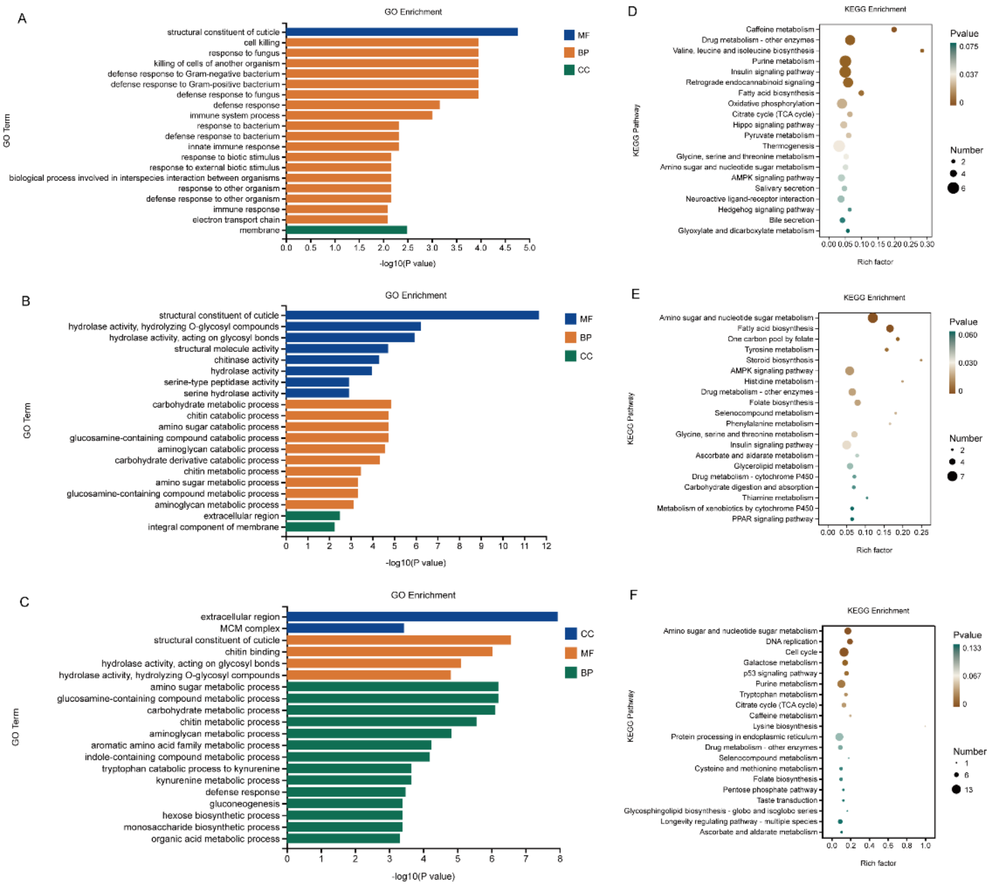

Figure S4 GO enrichment analysis and KEGG enrichment analysis of DEGs in *C. suppressalis*. (A) GO enrichment analysis of the treatment of T. (B) GO enrichment analysis of the treatment of M. (C) GO enrichment analysis of the treatment of B. (D) KEGG enrichment analysis of the treatment of T. (E) KEGG enrichment analysis of the treatment of M. (F) KEGG enrichment analysis of the treatment of B.

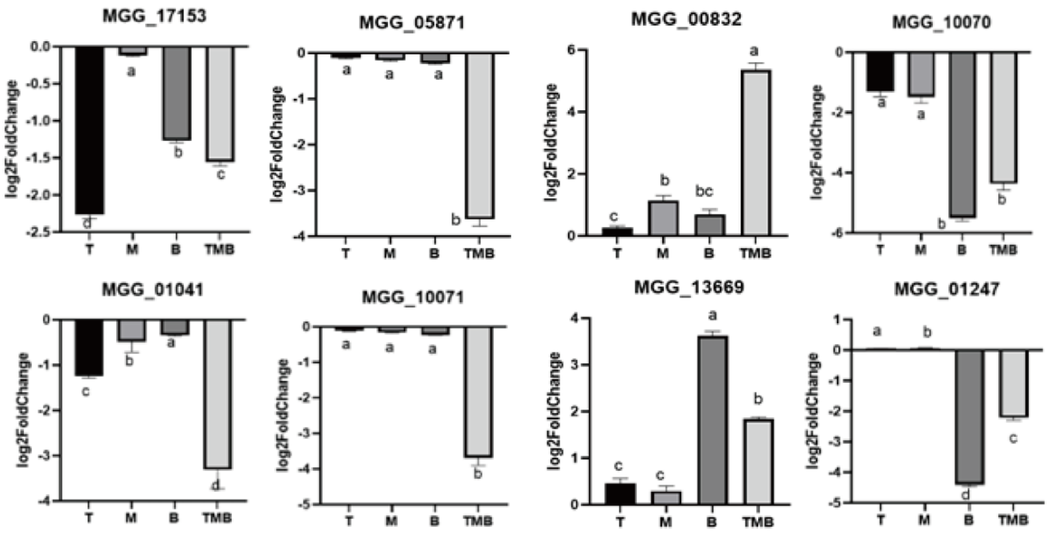

Figure S5 Validation of RNA-Seq results of *M. oryzae* using qRT-PCR.

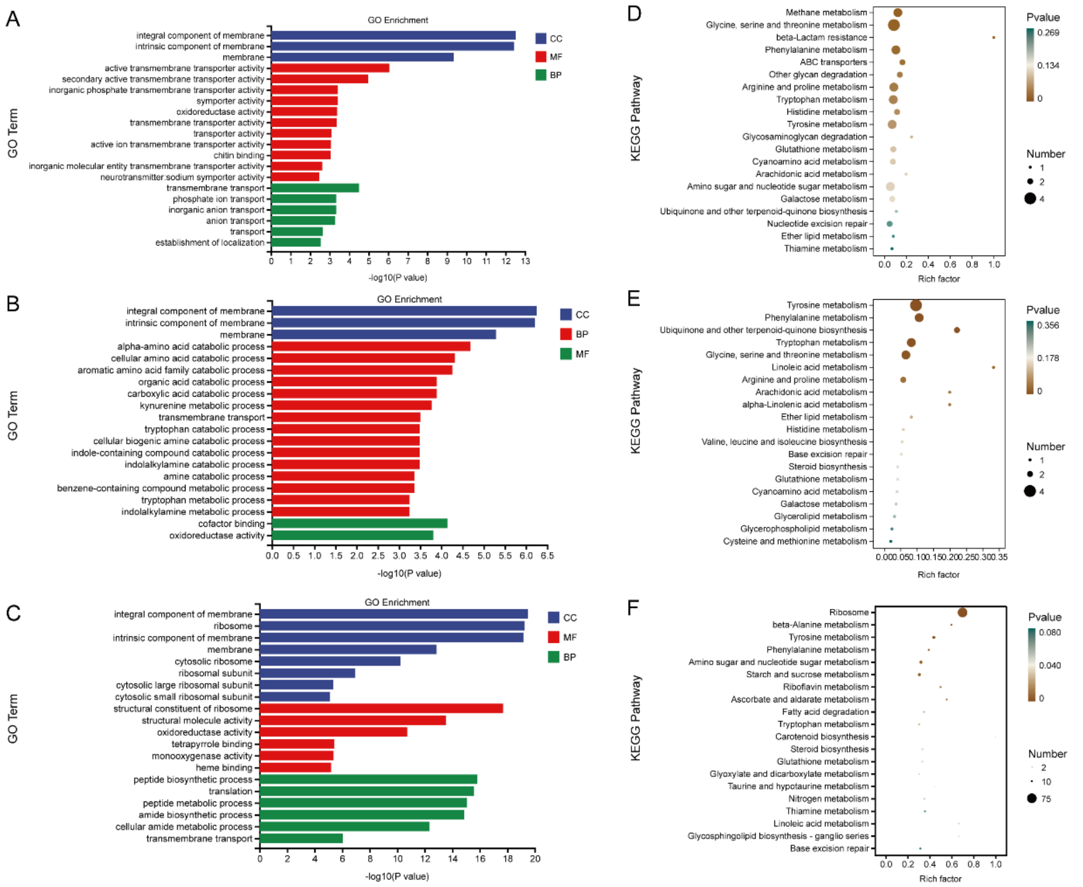

Figure S6 GO enrichment analysis and KEGG enrichment analysis of DEGs in M. oryzae.

(A) GO enrichment analysis of the treatment of T. (B) GO enrichment analysis of the treatment of M. (C) GO enrichment analysis of the treatment of B. (D) KEGG enrichment analysis of the treatment of T. (E) KEGG enrichment analysis of the treatment of M. (F) KEGG enrichment analysis of the treatment of B.

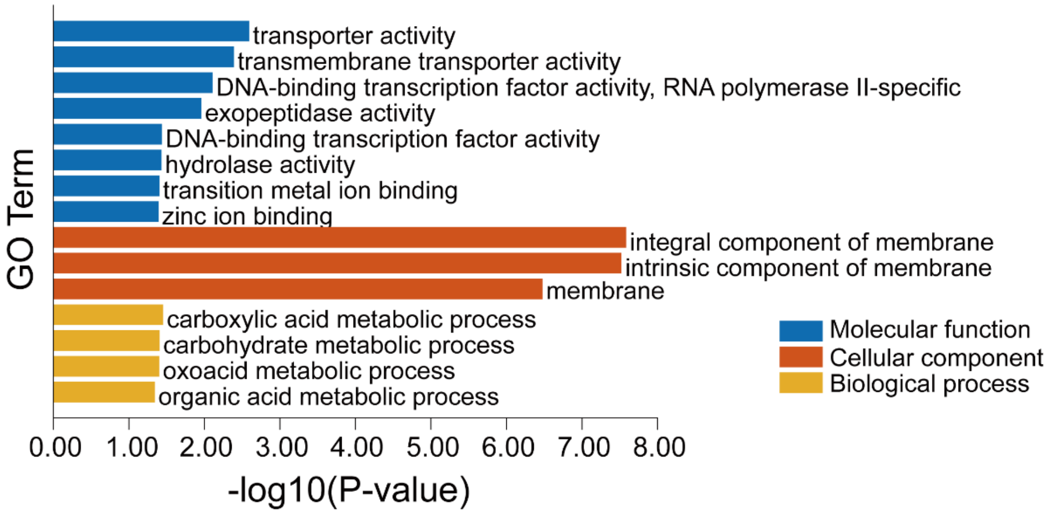

Figure S7 GO analysis of unique DEGs in TMB.

Table S1 PB design for *Trichoderma asperellum 10264* medium components.

Code	Factors	Levels		
		-1	0	1
A	maltose	10	20	30
B	galactose	10	20	30
C	skim milk powder	4	6	8
D	ammonium sulfate	0.5	1.0	1.5
E	magnesium sulfate	0.5	1.0	1.5
F	dipotassium hydrogen phosphate	0.5	1.0	1.5
G	Blank1	-1	0	1
H	Blank2	-1	0	1

Table S2 PB design for *Bacillus subtilis* S4-4-10 medium components.

Codes	Factors	Levels		
		-1	0	1
A	molasses	10	20	30
B	galactose	10	20	30
C	yeast powder	4	6	8
D	beef extract	4	6	8
E	ammonium sulfate	0.5	1.0	1.5
F	magnesium sulfate heptahydrate	0.5	1.0	1.5
G	potassium dihydrogen phosphate	0.5	1.0	1.5
H	Blank	-1	0	1

Table S3 PB design for *Metarhizoma anisopliae* 3.11962 medium components.

Codes	Factors	Levels		
		-1	0	1
A	sucrose	10	20	30
B	molasses,	10	20	30
C	soy peptone	4	6	8
D	beef extract	4	6	8
E	ammonium sulfate	0.5	1	1.5
F	magnesium sulfate heptahydrate	0.5	1	1.5
G	potassium dihydrogen phosphate	0.5	1	1.5
H	potassium dihydrogen phosphate	0.5	1	1.5
I	Blank	-1	0	1

Table S4 Center combination design for *Trichoderma* asperellum 10264 medium components.

Codes	Factors	Levels				
		-2	-1	0	1	2
A	potassium dihydrogen phosphate	0.6	0.7	0.8	0.9	1.0
B	Galactose	20	22	24	26	28
C	malt dust	12	14	16	18	20
D	ammonium sulphate	1.0	1.1	1.2	1.3	1.4

Table S5 Center combination design for Metarhizoma *anisopliae* 3.11962 medium components.

Codes	Factors	Levels				
		-2	-1	0	1	2
A	ammonium sulphate	0.1	0.4	0.7	1.0	1.3
B	molasses	12	18	24	30	36
C	Soy peptone	3.6	5.2	6.8	8.4	10.0
D	magnesium sulphate	0.5	0.7	0.9	1.1	1.3

Table S6 Primers of *C. suppressalis* larva used for qRT-PCR analysis

Gene ID	Forward (F)/Reverse (R)	sequence
evm_011424	F	TTGTTTGGTTTATGGAGCCGAGTG
evm_011424	R	CTTCCGCCAGGTTTGGTACTATTG
evm_003185	F	TATAGTCTCCTTCGGCGGCAAG
evm_003185	R	GTAGGCTTCGTTGGCAGTGATG
evm_010103	F	ACGGAGAGGCGAACTACACAG
evm_010103	R	GTTCCAGGACATTTTACCCCAACC
evm_001742	F	AGAGGTTGTGGAGGCTGGTTG
evm_001742	R	GACACTGCGTTGCGGTATCTG
evm_012682	F	TCAAACTCGGAAAAGCCACTGTC
evm_012682	R	CCTGTGAGTTACTGGTTGTCCTTG
evm_000739	F	AGCCTCATCGAGATACACATTTGC
evm_000739	R	CTCAACGCAGTAGCCGATATAAGC
evm_015191	F	TGGTGGCTGAACGAGGACTAC
evm_015191	R	TTCTTGCCTGTCAGTGTGTTAGTG
evm_008220	F	ATCCTAATCGGCAGTAGCAAACAAG
evm_008220	R	GCCAGGTCTTCCGTCTCCAC
evm_014213	F	GCGGCGTGATGACGAAGATC
evm_014213	R	TGGTTGTCCGAGTACGTGGTG
evm_013110	F	CGTCGCATTCACAGACACAGAG
evm_013110	R	GGATCTTCGGGTCGTCAAACTTG
evm_012682	F	TCAAACTCGGAAAAGCCACTGTC
evm_012682	R	CCTGTGAGTTACTGGTTGTCCTTG

Table S7 Primers of *M. oryzae* used for qRT-PCR analysis

Gene ID	Forward (F)/Reverse (R)	sequence
MGG_01247	F	CCAACCAGAACCGTTACTGTCAC
MGG_01247	R	AGGCGAAGAGGCAGGACTTG
MGG_17153	F	TTCACTCTCTGCTTTGGCTTTGG
MGG_17153	R	AGGCGTGATGTGGTACTGGTC
MGG_13669	F	CCGTTCTGTCCAAGTTCCTCAAG
MGG_13669	R	AGGCATCGTCGTAGGTCATCC
MGG_05871	F	TTGCCATTATCGGATTCGCTGTC
MGG_05871	R	TGTAATCGTCCCACCAGAGTCG
MGG_10071	F	TCTCATTCCAACTCAACAGCATAGC
MGG_10071	R	TGTGCAGAGGGCGGATAACG
MGG_01041	F	CCCACTACCTCCCACGACAAG
MGG_01041	R	TCATGGCACAAGAAGAGTCAATCG
MGG_10070	F	TGACGGAGACGCAGGACAAC
MGG_10070	R	TGCGGACCTCTCAGTCAACAC
MGG_02509	F	AGCGGAGCCAGGATGAGAATG
MGG_02509	R	GGGTGTAAGTAATGGGAGCAGTTTC
MGG_02235	F	GCCAAGAAGCCAGCGAGATATC
MGG_02235	R	CTGGAACAGCAGCGTGATGAC
MGG_00832	F	CTCGCTCTGGCTCAAGTTCAAG
MGG_00832	R	TTCGGCAATCTTCTCATCGGATATG
MGG_04628	F	CGCCGAGGAGGTGTACCAAG
MGG_04628	R	TGATGGAGTGGATGGACGAGTG

Table S8 Primers of Rice used for qRT-PCR analysis

Gene ID	Forward (F)/Reverse (R)	sequence
EF1-α	F	CTGTGCTGTCCTCATTATTG
EF1-α	R	AGGGTGAAAGCAAGAAGAGC
PR1	F	TGCTCAACAATATGCGAACC
PR1	R	TCATCCACCCACAACTGAAC
PAD4	F	AGAACAGCAAGCAGAATAGGAC
PAD4	R	CTTGAAGCAATCGTAGTAACCC
LOX2	F	TTGGAGGAAACAAAATCAAAGGGA
LOX2	R	TGGCACTAATGAGTTGGAAAGAAA
COI1	F	CCTAAGAGCAAACTTGACCCA
COI1	R	CAATGCCTCAGCAACTGTAAG
AOC	F	ACAAAAGGGGCACCAGC
AOC	R	CCAACTCTCAGTAGAAGGCAA
EDS5	F	AGAACACATAGCCAGCAAAGG
EDS5	R	GGAGATGACGGAGGAAGAAAG
NPR1	F	TTACTGATAAGGGCAAGAAGGCC
NPR1	R	AAAGTTCACAAAGAGCAGGATGG

Table S9 Evaluation of the effect of each factor in the Plackett-Burman design of medium components for *T. asperellum* 10264

Indicating pathogen	Factors	Effect	Coefficient	Coefficient standard error	T value	P value	Variance inflation factor
	Constant		55.86	3.71	15.04	0.000	
	A	-4.20	-2.10	3.71	-0.57	0.589	1.00
	B	11.37	5.68	3.71	1.53	0.170	1.00
Rhizoctonia solani	C	-30.95	-15.47	3.71	-4.17	0.004	1.00
	D	-7.66	-3.83	3.71	-1.03	0.337	1.00
	E	1.36	0.68	3.71	0.18	0.859	1.00
	F	-6.37	-3.18	3.71	-0.86	0.419	1.00
	Ct Pt		5.89	8.30	0.71	0.501	1.00
	Constant		52.64	3.24	16.26	0.000	
	A	-21.05	-10.53	3.24	-3.25	0.014	1.00
	B	21.28	10.64	3.24	3.29	0.013	1.00
Magnaporthe orizae	C	5.04	2.52	3.24	0.78	0.462	1.00
	D	18.58	9.29	3.24	2.87	0.024	1.00
	E	7.53	3.77	3.24	1.16	0.283	1.00
	F	-21.89	-10.94	3.24	-3.38	0.012	1.00
	Ct Pt		-34.07	7.24	-4.71	0.002	1.00
	Constant		31.65	3.09	10.24	0.000	
	A	6.67	3.33	3.09	1.08	0.317	1.00
	B	-1.38	-0.69	3.09	-0.22	0.830	1.00
Chilo suppressalis	C	-10.68	-5.34	3.09	-1.73	0.128	1.00
	D	10.17	5.09	3.09	1.65	0.144	1.00
	E	-0.01	-0.01	3.09	-0.00	0.999	1.00
	F	-9.03	-4.51	3.09	-1.46	0.188	1.00
	Ct Pt		10.41	6.91	1.51	0.176	1.00

Notes: A: maltose; B: galactose; C: skim milk powder; D: ammonium sulphate; E: magnesium sulphate; F: potassium dihydrogen phosphate

Table S10 Evaluation of the effect of each factor in the Plackett-Burman design of medium components for *B. subtilis* S4-4-10

Indicating pathogen	Factors	Effect	Coefficient	Coefficient standard error	T value	P value	Variance inflation factor
Rhizoctonia solani	Constant		76.095	0.817	93.18	0.000	
	A	2.972	1.486	0.817	1.82	0.143	1.00
	B	1.526	0.763	0.817	0.93	0.403	1.00
	C	4.635	2.318	0.817	2.84	0.047	1.00
	D	0.314	0.157	0.817	0.19	0.857	1.00
	E	-0.356	-0.178	0.817	-0.22	0.838	1.00
	F	-0.496	-0.248	0.817	-0.30	0.777	1.00
	G	1.201	0.600	0.817	0.74	0.503	1.00
	H	0.891	0.445	0.817	0.55	0.614	1.00
	J	0.460	0.230	0.817	0.28	0.792	1.00
	Ct Pt		-0.62	1.83	-0.34	0.751	1.00
Magnaporthe orizae	Constant		74.06	2.33	31.78	0.000	
	A	5.20	2.60	2.33	1.12	0.327	1.00
	B	1.52	0.76	2.33	0.33	0.760	1.00
	C	13.66	6.83	2.33	2.93	0.043	1.00
	D	9.32	4.66	2.33	2.00	0.116	1.00
	E	2.09	1.05	2.33	0.45	0.676	1.00
	F	-5.50	-2.75	2.33	-1.18	0.303	1.00
	G	-5.04	-2.52	2.33	-1.08	0.340	1.00
	H	1.21	0.61	2.33	0.26	0.807	1.00
	J	2.05	1.02	2.33	0.44	0.683	1.00
	Ct Pt		-10.23	5.21	-1.96	0.121	1.00
Chilo suppressalis	Constant		65.901	0.809	81.45	0.000	
	A	-6.426	-3.213	0.809	-3.97	0.017	1.00

B	19.654	9.827	0.809	12.15	0.000	1.00
C	-7.198	-3.599	0.809	-4.45	0.011	1.00
D	11.409	5.704	0.809	7.05	0.002	1.00
E	-15.487	-7.743	0.809	-9.57	0.001	1.00
F	11.386	5.693	0.809	7.04	0.002	1.00
G	-0.033	-0.016	0.809	-0.02	0.985	1.00
H	-0.871	-0.435	0.809	-0.54	0.619	1.00
J	3.142	1.571	0.809	1.94	0.124	1.00
Ct Pt		-4.86	1.81	-2.68	0.055	1.00

Notes: A: corn flour; B: molasses; C: galactose; D: yeast powder; E: soy peptone; F: beef powder; G: ammonium sulphate; H: magnesium sulphate; J: Potassium dihydrogen phosphate

Table S11 Evaluation of the effect of each factor in the Plackett-Burman design of medium components for *M. anisopliae* 3.11962

Indicating pathogen	Factors	Effect	Coefficient	Coefficient standard error	T value	P value	Variance inflation factor
	Constant		36.26	2.61	13.92	0.001	
	A	-14.74	-7.37	2.61	-2.83	0.066	1.00
	B	-39.18	-19.59	2.61	-7.52	0.005	1.00
	C	-17.73	-8.87	2.61	-3.40	0.042	1.00
	D	-10.74	-5.37	2.61	-2.06	0.131	1.00
Rhizoctonia	E	-1.04	-0.52	2.61	-0.20	0.855	1.00
solani	F	8.04	4.02	2.61	1.54	0.220	1.00
	G	16.00	8.00	2.61	3.07	0.055	1.00
	H	12.42	6.21	2.61	2.38	0.097	1.00
	J	-9.90	-4.95	2.61	-1.90	0.153	1.00
	K	6.79	3.40	2.61	1.30	0.283	1.00
	Ct Pt		45.45	5.83	7.80	0.004	1.00
	Constant		56.76	1.32	43.06	0.000	
	A	-26.12	-13.06	1.32	-9.91	0.002	1.00
	B	-23.96	-11.98	1.32	-9.09	0.003	1.00
	C	-15.54	-7.77	1.32	-5.89	0.010	1.00
	D	-17.61	-8.81	1.32	-6.68	0.007	1.00
Magnaporthe	E	8.46	4.23	1.32	3.21	0.049	1.00
orizae	F	0.92	0.46	1.32	0.35	0.751	1.00
	G	30.49	15.24	1.32	11.56	0.001	1.00
	H	13.53	6.77	1.32	5.13	0.014	1.00
	J	1.24	0.62	1.32	0.47	0.669	1.00
	K	-11.13	-5.57	1.32	-4.22	0.024	1.00
	Ct Pt		29.03	2.95	9.85	0.002	1.00

	Constant		37.80	2.58	14.66	0.001	
	A	-16.07	-8.04	2.58	-3.12	0.053	1.00
	B	-22.02	-11.01	2.58	-4.27	0.024	1.00
	C	-22.02	-11.01	2.58	-4.27	0.024	1.00
	D	-16.07	-8.04	2.58	-3.12	0.053	1.00
Chilo	E	0.60	0.30	2.58	0.12	0.915	1.00
suppressalis	F	-6.55	-3.27	2.58	-1.27	0.294	1.00
	G	-8.93	-4.46	2.58	-1.73	0.182	1.00
	H	19.64	9.82	2.58	3.81	0.032	1.00
	J	14.88	7.44	2.58	2.89	0.063	1.00
	K	-12.50	-6.25	2.58	-2.42	0.094	1.00
	Ct Pt		26.49	5.76	4.60	0.019	1.00

Notes: A: sucrose; B: molasses; C: galactose; D: soy peptone; E: tryptone; F: beef powder; G: ammonium sulphate; H: magnesium sulphate; J: Potassium dihydrogen phosphate; K: dipotassium hydrogen phosphate

Table S12 The steepest climbing test design and results of *T. asperellum* 10264 and *M. anisopliae* 3.11962.

	No.	Potassium dihydrogen phosphate (g/L)	Galactose (g/L)	Malt dust (g/L)	Ammonium sulphate (g/L)	Inhibition rate against *M. oryzae*
T. asperellum 10264	1	1.1	18	22	0.9	24.17±10.43
	2	1	20	20	1	8.55±13.86
	3	0.9	22	18	1.1	45.84±3.93
	4	0.8	24	16	1.2	55.69±13.45
	5	0.7	26	14	1.3	43.44±8.88
	6	0.6	28	12	1.4	36.47±17.38
	No.	Ammonium sulphate (g/L)	Molasses (g/L)	Soy peptone (g/L)	Magnesium sulphate (g/L)	Inhibition rate against *M. oryzae*
M. anisopliae 3.11962	1	0.7	24	6.8	0.9	88.53±1.27
	2	1.0	20	6	1.0	87.48±1.27
	3	1.3	16	5.2	1.1	85.07±2.82
	4	1.6	12	4.4	1.2	81.8±4.76
	5	1.9	8	3.6	1.3	81.35±3.23
	6	2.2	4	2.8	1.4	35.53±1.34

Table S13 Types and contents of biomarkers in different culture filtrates

Biomarkers	T	M	B	TMB
6pp (ng/ml)	203.67	308.40	244.06	189.66
alamethicin (ppb)	0.30	0.21	0.37	0.26
hydrophobin (μg/L)	67.78	82.40	96.92	60.74
IAA (μg/L)	88.14	97.61	79.09	81.33
gibberellin (pg/ml)	517.05	468.17	569.44	444.90
SA (pmol/L)	1531.05	1290.80	1459.33	1457.42
calmodulin (μg/L)	10.42	9.30	9.23	7.80
chitinase (pg/ml)	160.58	171.82	184.86	177.19
glucanase (ng/L)	388.72	408.50	447.19	418.66
cellulase (pg/mL)	116.49	136.08	118.09	124.02
oxalic acid (mg/l)	12.9	643.3	137.8	85.4
acetic acid (mg/l)	206.6	2231.1	199.5	1120.2
citric acid (mg/l)	136.7	3162	667.2	N.D.
phosphoserine	9410	114316	62037	24236
taurine	3783	60593	18364	10434
aspartic acid	N.D.	14290	14558	N.D.
threonine	N.D.	4359	3090	1152
serine	N.D.	4383	3285	1581
glutamate	N.D.	7088	30405	8304
glycine	N.D.	7459	1475	2410
alanine	N.D.	8933	13932	3094
valine	1592	13769	7909	3728
cysteine	2064	11302	2290	733
methionine	N.D.	3508	2256	906
isoleucine	N.D.	2618	7627	2392
leucine	N.D.	5944	25621	5806
tyrosine	N.D.	6309	16704	3676
phenylalanine	233	23838	97236	16454
aminobutyric acid	396	16997	1631	485
ornithine	N.D.	5364	N.D.	1242
lysine	N.D.	8101	29209	8657
histidine	N.D.	1251	2124	1063
arginine	N.D.	6000	7813	2910

Notes: "N. D." represents "not undetected".

Table S14 Summary of RNA-seq reads of *C. suppressalis* treated in different metabolites.

Sample	Raw reads	Clean reads	Q30 (%)	GC content(%)	Total mapped
T1	40026798	39520638	94.42	47.76	36975973 (93.56%)
T2	43461494	42932270	94.62	47.64	40071329 (93.34%)
T3	40010886	39465096	94.2	47.49	36819130 (93.30%)
M1	48144046	47578070	94.54	47.75	44340801 (93.20%)
M2	48060508	47395156	94.18	47.28	44100042 (93.05%)
M3	40810142	40253508	94.02	47.65	37479111 (93.11%)
B1	41171066	40655744	94.65	47.53	37926379 (93.29%)
B2	43220470	42720948	94.63	48.28	39913080 (93.43%)
B3	42136804	41586282	94.44	47.79	38812846 (93.33%)
TMB1	43208020	42659602	94.64	49.31	39988465 (93.74%)
TMB2	43261880	42704416	94.61	49.13	40005996 (93.68%)
TMB3	43473198	42934682	94.77	49.23	40228364 (93.70%)
CK1	42854056	42326220	94.57	47.95	39483270 (93.28%)
CK2	46603754	46034486	94.66	47.78	42989644 (93.39%)
CK3	44815836	44205014	94.31	47.71	41327477 (93.49%)

Table S15 Summary table of major DEGs related to immunity in *C. suppressalis* exposed to treatment compared to untreated groups.

Gene Name	Function	Expression (Log2FC)			
		CK VS T	CK VS M	CK VS B	CK VS TMB
evm_013596	Cecropin-D-like peptide	1.198	-0.515	0.074	-1.773
evm_014802	Attacin-A	2.782	0.680	1.317	-2.002
evm_006288	Cecropin-D-like peptide	-1.001	-0.866	-1.173	-1.909
evm_010951	chitinase	-0.757	-3.228	-3.709	-0.281
evm_015633	chitinase 4	0.113	-1.689	-1.809	-1.104
evm_008789	chitinase 2	-0.024	-1.134	-0.587	-0.789
evm_008793	chitinase 11	-1.670	-1.966	-0.557	-0.793
evm_009925	Endochitinase	-0.801	-2.975	-3.906	-0.073
evm_011180	Phenoloxidase subunit 1	-0.246	-0.782	-1.231	-0.776
evm_014803	Defense protein 3	-0.731	-1.740	-1.974	-3.233
evm_015343	Attacin-A	-0.944	-1.386	-1.264	-2.656
evm_009766	Attacin-A	-0.732	-1.455	-1.225	-1.718
evm_010335	Growth arrest and DNA damage-inducible protein GADD45 alpha	0.528	-0.468	-1.044	-1.480
evm_010500	ATP-dependent DNA helicase PIF1	-0.216	-0.034	-0.608	-2.922
evm_001408	N-glycosylase/DNA lyase	0.055	-0.565	0.126	-1.739

Table S16 Summary of RNA-seq reads of *M. oryzae* treated in different metabolites.

Sample	Raw reads	Clean reads	Q30 (%)	GC content(%)	Total mapped
T1	41464210	40540352	94.41	54.92	40023112 (98.72%)
T2	47645164	46695872	94.96	54.89	46137128 (98.80%)
T3	49211114	48139826	94.72	54.75	47471554 (98.61%)
M1	49213172	48137396	94.68	54.84	47457798 (98.59%)
M2	50323360	49248574	94.72	54.75	48548905 (98.58%)
M3	40669884	39722224	94.57	54.92	39153394 (98.57%)
B1	45442192	44452874	94.65	55.33	43506894 (97.87%)
B2	47687510	46700464	94.8	55.31	45915987 (98.32%)
B3	47484766	46383082	94.62	55.41	45581211 (98.27%)
TMB1	49519632	48466476	94.78	55.3	47758351 (98.54%)
TMB2	43880066	42955558	94.77	55.3	42340072 (98.57%)
TMB3	45876120	44911762	94.79	55.31	44256467 (98.54%)
CK1	43907556	42960882	94.69	54.85	42275162 (98.40%)
CK2	46614706	45632390	94.72	54.7	44832531 (98.25%)
CK3	48513514	47507122	94.75	54.68	46857334 (98.63%)

Table S17 Summary of major DEGs related to membrane in *M. oryzae* exposed to treatment compared to untreated groups

Gene ID	Function	log_2FC			
		T	M	B	TMB
MGG_03266	hypothetical protein	-0.390	-0.200	-0.825	-1.097
MGG_07980	metabolite transporter	-0.319	0.140	-0.798	-1.174
MGG_04230	pantothenate transporter FEN2	-0.257	-0.261	-0.398	-1.046
MGG_04225	quinate permease	-0.899	0.194	-0.819	-2.016
MGG_01191	hypothetical protein	0.007	0.344	0.801	1.024
MGG_16233	hypothetical protein	0.020	0.496	0.840	1.430
MGG_08815	hypothetical protein	0.793	-0.100	0.749	1.066
MGG_07761	hypothetical protein	0.231	0.196	0.872	1.516
MGG_15354	hypothetical protein	-0.014	0.192	-0.701	-3.332
MGG_04362	hypothetical protein	0.016	0.214	0.897	1.451
MGG_05451	1-acyl-sn-glycerol-3-phosphate acyltransferase 2	0.270	0.236	0.725	1.026
MGG_03395	hypothetical protein	0.755	0.314	0.867	1.590
MGG_15239	hypothetical protein	-0.393	-0.431	-0.523	-1.312
MGG_04554	hypothetical protein	0.255	0.858	0.910	1.115
MGG_03102	vacuolar amino acid transporter 6	-0.714	-0.266	-0.654	-1.426
MGG_16424	alkaline phytoceramidase	-0.054	0.095	-0.957	-1.491
MGG_01390	MFS hexose transporter	-0.583	-0.688	-0.571	-1.068
MGG_04656	hypothetical protein	0.160	0.332	0.793	1.100
MGG_01051	PET8 related protein	0.812	-0.292	-0.929	-1.051
MGG_06738	G-protein coupled receptor	0.715	-0.010	-0.208	1.009
MGG_07334	hypothetical protein	0.122	-0.001	0.756	1.256
MGG_08207	hypothetical protein	-0.632	-0.321	-0.973	-1.079
MGG_07233	hypothetical protein	-0.083	-0.361	-0.569	-1.145
MGG_10865	hypothetical protein	-0.669	-0.397	-0.701	-1.471

MGG_05124	hypothetical protein	0.156	0.130	0.783	1.182
MGG_07546	alpha-glucosides permease MPH2/3	0.676	0.260	0.298	2.340
MGG_08501	hypothetical protein	-0.825	0.154	2.513	-2.629
MGG_05595	spermidine/putrescine import ATP-binding protein potA	0.901	-0.050	0.293	2.101
MGG_05233	hypothetical protein	-0.165	0.052	0.626	1.142
MGG_09827	sugar transporter family protein	-0.579	-0.945	-0.774	-1.666
MGG_05386	hypothetical protein	1.436	0.676	-1.864	2.550
MGG_05139	hypothetical protein	-0.984	-0.723	-0.284	-1.477
MGG_06584	hypothetical protein	0.334	0.215	0.991	1.523
MGG_08761	peptidase S41 family protein	0.797	-0.120	0.706	1.623
MGG_07428	allantoate permease	-0.478	-0.057	-0.628	-2.424
MGG_10474	hypothetical protein	0.135	0.484	0.958	1.636
MGG_10598	transporter protein	-0.660	-0.149	0.969	1.575
MGG_01996	hypothetical protein	0.772	0.474	0.245	1.539
MGG_10492	mitochondrial carnitine carrier	0.345	0.055	0.896	1.010
MGG_09268	hypothetical protein	0.025	0.098	0.569	1.062
MGG_10212	hypothetical protein	-0.612	0.198	0.153	-2.925
MGG_04804	hypothetical protein	0.014	0.259	0.739	1.040
MGG_00447	brefeldin A resistance protein	0.613	-0.419	0.685	1.228
MGG_02982	averantin oxidoreductase	0.288	-0.175	-0.699	-1.080
MGG_02974	mitochondrial carrier protein RIM2	0.288	0.275	-0.754	-1.042
MGG_03821	cytochrome P450 3A19	-0.325	-0.507	1.573	1.956
MGG_00799	peptide-N4-(N-acetyl-beta-glucosaminyl) asparagine amidase A	0.023	-0.048	-0.481	-1.243
MGG_11529	hypothetical protein	0.324	0.484	0.870	1.074
MGG_13948	hypothetical protein	0.695	0.095	0.686	1.195
MGG_02801	hypothetical protein	-0.317	-0.068	-0.200	-1.026
MGG_06167	phytase	0.399	0.047	0.389	1.254

MGG_09309	ER-derived vesicles protein ERV29	-0.199	-0.014	-0.857	-1.061
MGG_07287	lysophospholipase 3	0.221	0.207	0.461	1.302
MGG_11860	NACHT and WD domain-containing protein	-0.931	-0.383	-0.966	-1.214
MGG_09341	LOW QUALITY PROTEIN: integral membrane protein	0.630	1.393	2.100	2.993
MGG_01859	hypothetical protein	-0.290	0.166	-0.876	-1.052
MGG_01612	hypothetical protein	-0.116	0.470	0.548	1.152
MGG_12848	hypothetical protein	0.134	0.372	-0.021	2.102
MGG_13934	hypothetical protein	-0.726	-0.124	-0.667	-2.078
MGG_11754	heavy metal tolerance protein	0.106	-0.001	-0.922	-1.080
MGG_01729	hypothetical protein	-0.304	0.135	0.880	1.247
MGG_00150	hypothetical protein	-0.160	0.095	0.893	1.393
MGG_04511	MFS transporter	0.608	-0.624	0.650	1.164
MGG_17825	arabinose-proton symporter	0.187	-0.302	-0.924	-1.640
MGG_15887	hypothetical protein	-0.300	0.093	0.853	1.382
MGG_15409	hypothetical protein	0.517	0.475	-0.161	1.455
MGG_04980	hypothetical protein	0.021	-0.082	-0.927	-1.015
MGG_12388	hypothetical protein	0.012	0.126	0.827	1.438
MGG_03769	hypothetical protein	-0.287	0.282	0.899	1.027
MGG_10057	hypothetical protein	0.109	0.179	0.905	1.079
MGG_04525	hypothetical protein	0.180	-0.125	-0.987	-1.528
MGG_02582	hypothetical protein	0.185	-0.335	-0.678	-1.121
MGG_02464	vacuolar amino acid transporter 3	0.401	-0.019	0.540	1.149
MGG_13334	general amino acid permease AGP2	-0.885	0.620	-0.686	-3.690
MGG_14548	integral membrane protein	0.127	-0.060	0.770	1.312
MGG_00158	purine-cytosine permease FCY21	-0.246	0.017	-0.815	1.028
MGG_02646	hypothetical protein	0.016	0.485	0.531	1.192
MGG_13798	hypothetical protein	0.454	0.527	0.291	1.064

MGG_13797	hypothetical protein	0.286	0.561	0.128	1.025
MGG_10051	hypothetical protein	-0.315	-0.168	-0.887	-1.262
MGG_03724	CLAP1	0.217	-0.016	0.944	1.135
MGG_04852	phospholipid-transporting ATPase 1	0.259	0.416	0.541	1.035
MGG_11357	chloride channel protein 3	0.443	0.010	0.398	1.744
MGG_15953	hypothetical protein	0.803	0.253	1.189	2.001
MGG_05929	hexose transporter protein	-0.945	-0.299	0.868	1.009

Table S18 Summary of major DEGs related to protein/ transcription factor in *M. oryzae* exposed to treatment compared to untreated groups

Gene ID	function	Log_2FC			
		T	M	B	TMB
MGG_07980	metabolite transporter	-0.319	0.140	-0.798	-1.174
MGG_04225	quinate permease	-0.899	0.194	-0.819	-2.016
MGG_01390	MFS hexose transporter	-0.583	-0.688	-0.571	-1.068
MGG_07233	hypothetical protein	-0.083	-0.361	-0.569	-1.145
MGG_07546	alpha-glucosides permease MPH2/3	0.676	0.260	0.298	2.340
MGG_05595	spermidine/putrescine import ATP-binding protein potA	0.901	-0.050	0.293	2.101
MGG_05233	hypothetical protein	-0.165	0.052	0.626	1.142
MGG_09827	sugar transporter family protein	-0.579	-0.945	-0.774	-1.666
MGG_10492	mitochondrial carnitine carrier	0.345	0.055	0.896	1.010
MGG_00447	brefeldin A resistance protein	0.613	-0.419	0.685	1.228
MGG_02974	mitochondrial carrier protein RIM2	0.288	0.275	-0.754	-1.042
MGG_11754	heavy metal tolerance protein	0.106	-0.001	-0.922	-1.080
MGG_17825	arabinose-proton symporter	0.187	-0.302	-0.924	-1.640
MGG_02464	vacuolar amino acid transporter 3	0.401	-0.019	0.540	1.149
MGG_13334	general amino acid permease AGP2	-0.885	0.620	-0.686	-3.690
MGG_00158	purine-cytosine permease FCY21	-0.246	0.017	-0.815	1.028
MGG_03724	CLAP1	0.217	-0.016	0.944	1.135
MGG_04852	phospholipid-transporting ATPase 1	0.259	0.416	0.541	1.035
MGG_11357	chloride channel protein 3	0.443	0.010	0.398	1.744
MGG_05929	hexose transporter protein	-0.945	-0.299	0.868	1.009
MGG_06312	C6 zinc finger domain-containing protein	0.386	0.221	0.828	1.032
MGG_06550	hypothetical protein	-0.763	-0.265	0.303	1.202
MGG_05459	hypothetical protein	-0.281	-0.211	-0.218	-1.022

MGG_07830	hypothetical protein	0.587	0.051	0.513	1.319
MGG_02089	hypothetical protein	2.017	0.591	0.588	2.384
MGG_02962	C6 zinc finger domain-containing protein	0.150	0.150	0.776	1.030
MGG_14816	hypothetical protein	-0.214	-0.092	-0.960	-1.562
MGG_10212	hypothetical protein	-0.612	0.198	0.153	-2.925
MGG_06832	hypothetical protein	-0.718	-0.361	-0.351	-1.032
MGG_02226	hypothetical protein	-0.427	-0.316	-0.777	-1.046
MGG_08711	carboxypeptidase A2	-0.358	-0.325	-0.824	-1.210
MGG_08785	X-Pro dipeptidyl-peptidase protein	0.019	0.136	-0.953	-1.122
MGG_09716	carboxypeptidase A2 precursor	-0.327	-0.420	-0.757	-1.168
MGG_01863	aminopeptidase Y	-0.383	-0.274	-0.750	-1.333
MGG_02309	carboxypeptidase S1	-0.149	0.442	-1.093	-1.547
MGG_04499	exoglucanase-6A	-0.830	-0.621	-0.853	-1.330
MGG_07646	alpha-glucuronidase	-1.349	-0.882	-1.302	-1.845
MGG_05340	hypothetical protein	-0.538	-0.276	-0.936	-1.103
MGG_06780	alpha-L-fucosidase 1	-0.190	-0.191	-0.976	-1.026
MGG_16424	alkaline phytoceramidase	-0.054	0.095	-0.957	-1.491
MGG_06750	hypothetical protein	0.225	0.529	0.906	1.918
MGG_09404	feruloyl esterase B	-0.167	-0.061	-0.449	-1.081
MGG_06493	beta-glucanase	-0.487	-0.626	-0.662	-1.171
MGG_09664	beta-mannosidase	-0.129	-0.086	-0.429	-1.127
MGG_08330	dienelactone hydrolase	-0.100	-0.122	-0.573	-1.446
MGG_01812	amidohydrolase	-0.406	-0.242	-0.160	-1.068
MGG_01800	flap structure-specific endonuclease	0.435	0.453	-0.491	-1.015
MGG_05596	hydrolase	-0.561	-0.624	0.604	1.271
MGG_04042	leucyl-tRNA synthetase	-0.382	-0.076	-0.308	-1.219
MGG_08761	peptidase S41 family protein	0.797	-0.120	0.706	1.623
MGG_08881	hypothetical protein	-0.203	-0.325	-0.881	-1.644

MGG_03070	epoxide hydrolase domain-containing protein	-0.059	-0.094	-0.331	-1.073
MGG_09249	peptidase S9 prolyl oligopeptidase active site-containing protein	0.543	0.099	0.876	1.195
MGG_06167	phytase	0.399	0.047	0.389	1.254
MGG_07287	lysophospholipase 3	0.221	0.207	0.461	1.302
MGG_11860	NACHT and WD domain-containing protein	-0.931	-0.383	-0.966	-1.214
MGG_02812	1-aminocyclopropane-1-carboxylate deaminase	0.352	0.105	0.680	1.389
MGG_10083	endoglucanase 3	-0.439	-0.292	-0.868	-1.178
MGG_07920	arylsulfatase	2.353	1.328	0.120	3.376
MGG_00296	glycosyl hydrolase	-0.357	-0.161	0.661	1.017
MGG_15760	hypothetical protein	0.708	0.441	-0.858	-1.109
MGG_02529	hypothetical protein	-0.198	-0.207	-0.773	-1.940
MGG_01433	elongation factor G	0.119	0.288	-0.674	-1.092
MGG_09272	beta-glucosidase 1	0.057	-0.346	-0.871	-2.088
MGG_10275	hypothetical protein	-0.321	-0.382	-0.445	-1.282

Table S19 Summary of major DEGs related to metabolic process in *M. oryzae* exposed to treatment compared to untreated groups

Gene ID	function	log2FC			
		T	M	B	TMB
MGG_02125	branched-chain-amino-acid aminotransferase 1	0.137	-0.199	0.921	1.190
MGG_03096	fructose-bisphosphate aldolase	-0.104	-0.229	-0.464	-1.508
MGG_05275	glutamyl-tRNA synthetase	-0.231	-0.123	-0.369	-1.128
MGG_03094	triosephosphate isomerase 2	-0.153	-0.215	0.046	-1.064
MGG_08450	asparagine synthase	-0.812	-0.092	-0.028	1.050
MGG_07782	catabolic 3-dehydroquinase	-0.207	-0.199	0.064	-1.007
MGG_04042	leucyl-tRNA synthetase	-0.382	-0.076	-0.308	-1.219
MGG_13512	aspartyl-tRNA synthetase	-0.146	-0.044	-0.643	-1.126
MGG_10492	mitochondrial carnitine carrier	0.345	0.055	0.896	1.010
MGG_01687	3-hydroxyisobutyrate dehydrogenase	-0.650	-0.847	0.086	-1.309
MGG_04499	exoglucanase-6A	-0.830	-0.621	-0.853	-1.330
MGG_07646	alpha-glucuronidase	-1.349	-0.882	-1.302	-1.845
MGG_06780	alpha-L-fucosidase 1	-0.190	-0.191	-0.976	-1.026
MGG_06493	beta-glucanase	-0.487	-0.626	-0.662	-1.171
MGG_09664	beta-mannosidase	-0.129	-0.086	-0.429	-1.127
MGG_08881	hypothetical protein	-0.203	-0.325	-0.881	-1.644
MGG_10083	endoglucanase 3	-0.439	-0.292	-0.868	-1.178
MGG_12025	aldose 1-epimerase	0.131	0.207	0.525	1.312
MGG_09272	beta-glucosidase 1	0.057	-0.346	-0.871	-2.088
